# Supplementary material for: Genome landscape and genetic architecture of recombination in domestic goats (Capra hircus)
Source: Genet Sel Evol. 2025 Oct 16;57:57. doi: 10.1186/s12711-025-01001-0 (PMC12532432; doi:10.1186/s12711-025-01001-0)
Supplement: Supplementary file 1 — Additional file 1: Figure S1. Position of (b)ovine (top) and goat (bottom) chip SNPs on both the RH map and the ARS1 assembly, on chromosomes 2 and 18. Goat SNPs have been filtered using the longest increasing subsequence algorithm, and only remaining SNPs have been represented. Figure S2. Crossover distribution on the 35-45 Mb area of chromosome 1. Red lines = position of conserved synteny breaks. Figure S3. Additive SNP and sample call-rate cumulative distributions. Left: SNP call-rate; right: sample call-rate. Black line: chosen threshold=0.01. Figure S4. Distribution of the distance between two crossovers per chromosome per meiosis. Red line: chosen threshold=5Mb. Figure S5. Density distribution of the Maximum Likelihood estimates of recombination rates on 1Mb intervals of the genome. Black: gamma law; extracted parameters α=3.08 & β=2.20. Figure S6. Distribution of crossover detection interval sizes. Figure S7. Distribution of all permuted recombination rate log ratio for all genomic intervals. Figure S8. SNP distribution of sequencing and variant calling quality parameters. A: DP parameter; B: FS parameter; C: VQSLOD parameter. Figure S9. R² evolution according to allele frequency. (A) Before genotype imputation. Mean R²=0.928. (B) After genotype imputation. Mean R²=0.990. Figure S10. Genotype concordance according to allele frequency. (A) Before genotype imputation. Mean genotype concordance=0.974. (B) After genotype imputation. Mean genotype concordance=0.997. Figure S11. Distribution the proportion of the autosomal genome informative for crossovers in parents (M=males vs F=females). Figure S12. Comparison of recombination map size estimates between populations. Confidence interval = μ±2×standard error. Figure S13. Distribution of genome-wide recombination rates (GRR) in meioses. Figure S14. Distribution of intra-chromosomal shuffling in meioses. Figure S15. Comparison of intra-chromosomal shuffling estimates between populations. Confidence interval = μ±2×st [file 12711_2025_1001_MOESM1_ESM.pdf]

Additional File 1

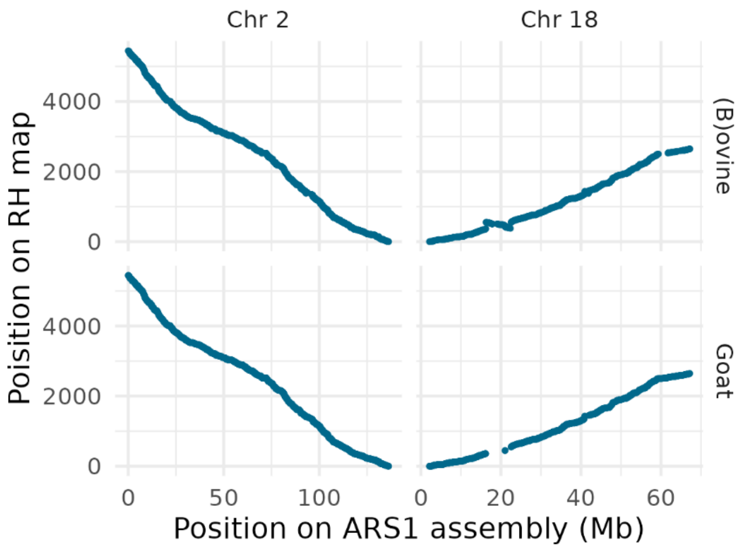

Figure S1: Position of (b)ovine (top) and goat (bottom) chip SNPs on both the RH map and the ARS1 assembly, on chromosomes 2 and 18. Goat SNPs have been filtered using the longest increasing subsequence algorithm, and only remaining SNPs have been represented.

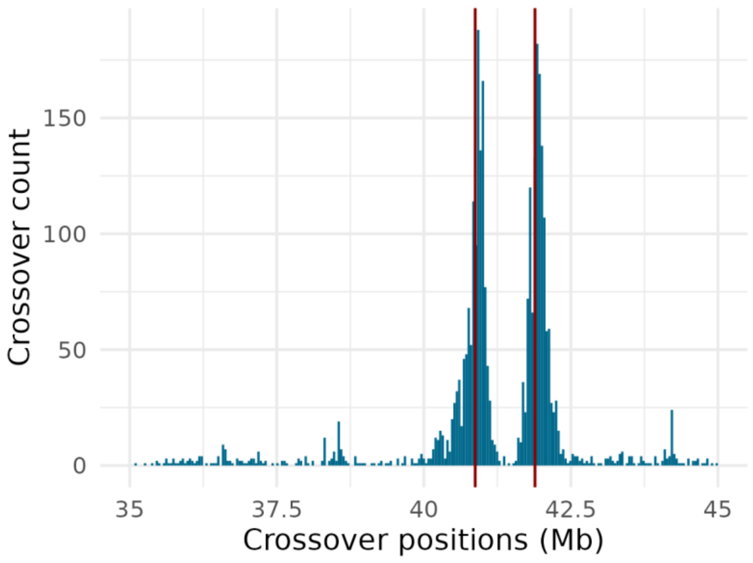

Figure S2 : Crossover distribution on the 35-45 Mb area of chromosome 1. Red lines = position of conserved synteny breaks.

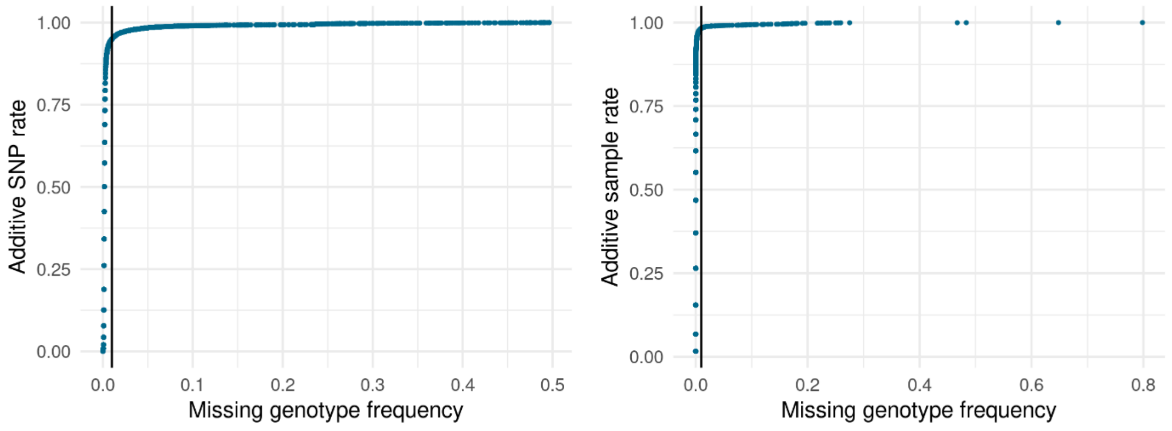

Figure S3: Additive SNP and sample call-rate cumulative distributions. Left: SNP call-rate; right: sample call-rate. Black line: chosen threshold=0.01.

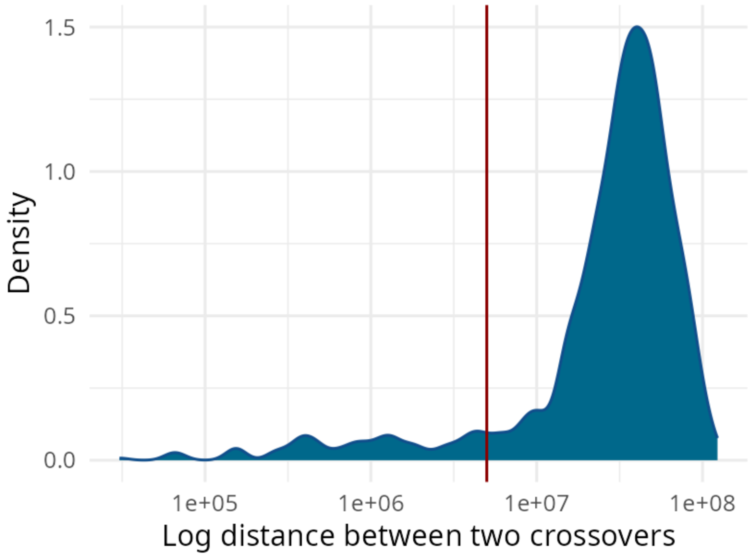

Figure S4: Distribution of the distance between two crossovers per chromosome per meiosis.

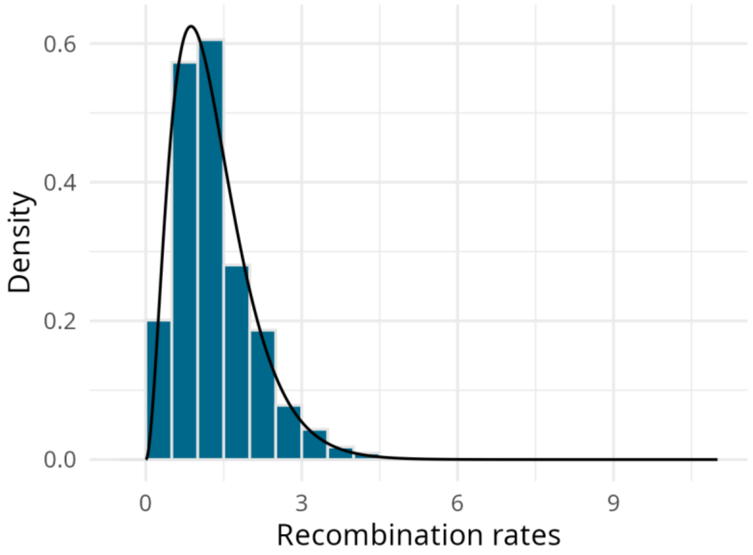

Figure S5: Density distribution of the Maximum Likelihood estimates of recombination rates on 1Mb intervals of the genome. Black: gamma law; extracted parameters α=3.08 & β=2.20.

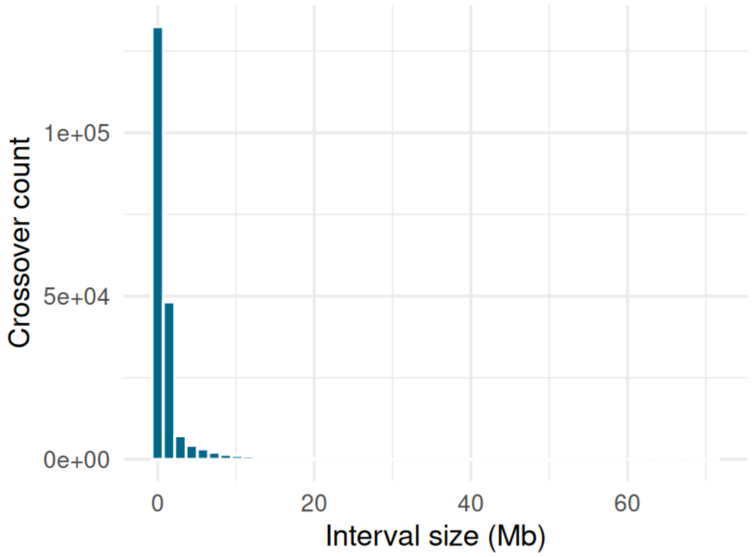

Figure S6: Distribution of crossover detection interval sizes.

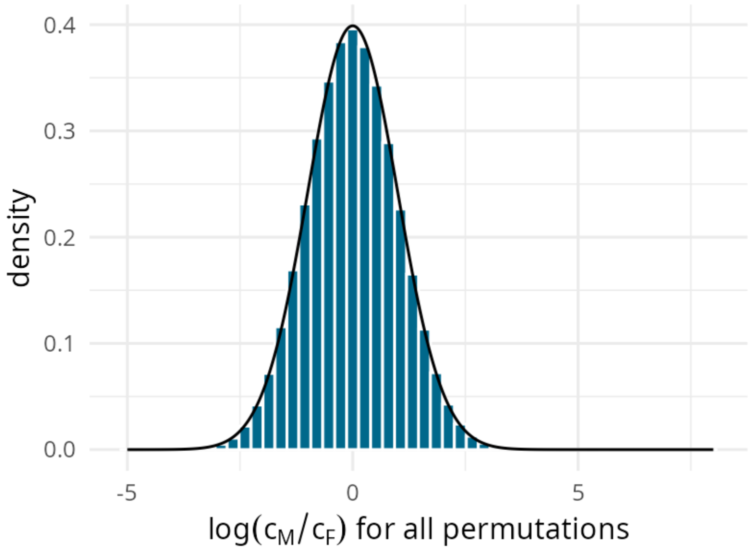

Figure S7: Distribution of all permuted recombination rate log ratio for all genomic intervals.

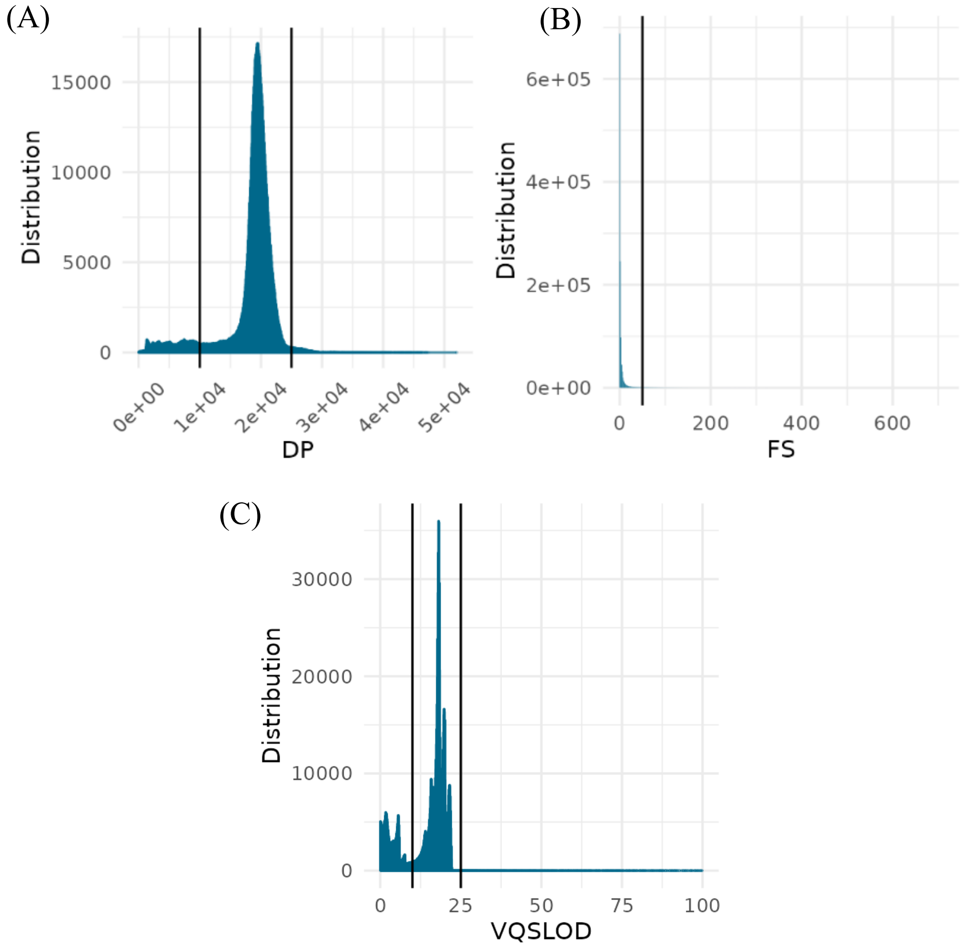

Figure S8: SNP distribution of sequencing and variant calling quality parameters. A: DP parameter; B: FS parameter; C: VQSLOD parameter.

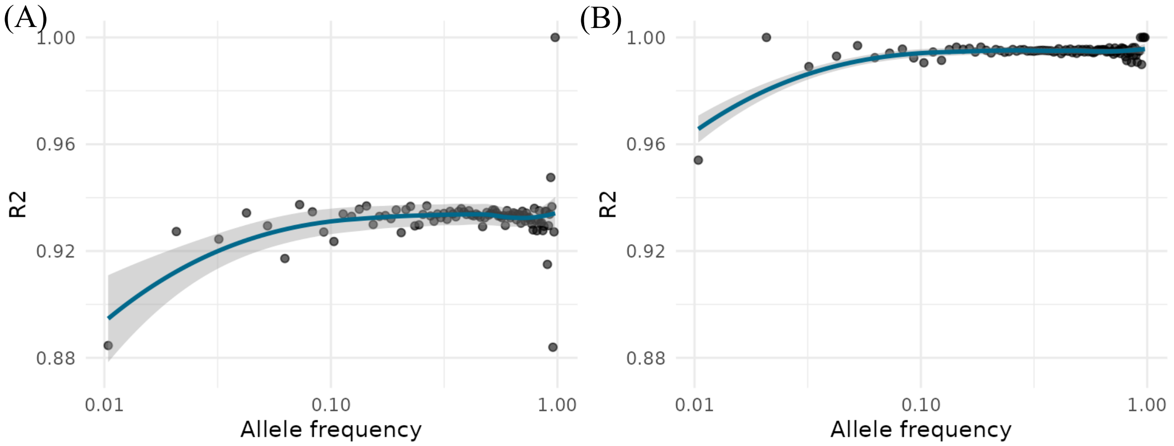

Figure S9: R^2 evolution according to allele frequency. (A) Before genotype imputation. Mean R^2=0.928. (B) After genotype imputation. Mean R^2=0.990.

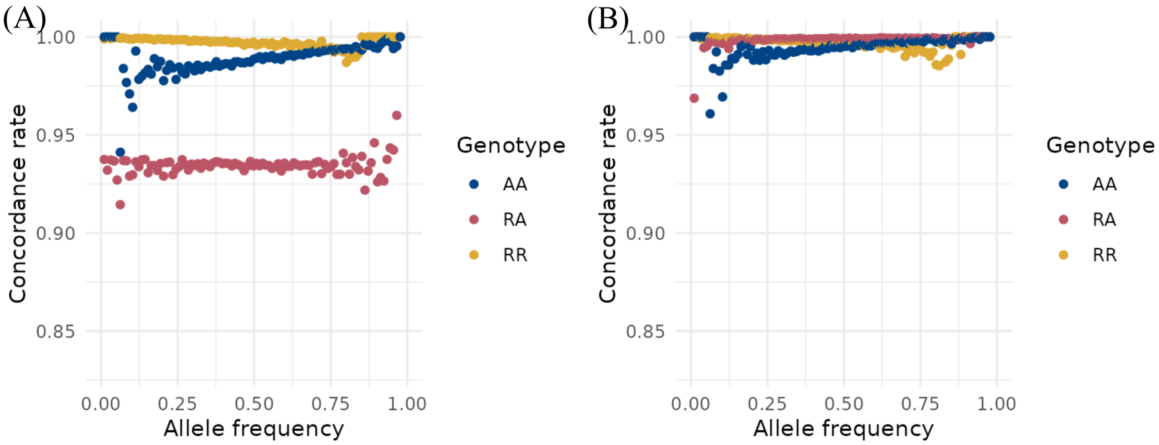

Figure S10: Genotype concordance according to allele frequency. (A) Before genotype imputation. Mean genotype concordance=0.974. (B) After genotype imputation. Mean genotype concordance=0.997.

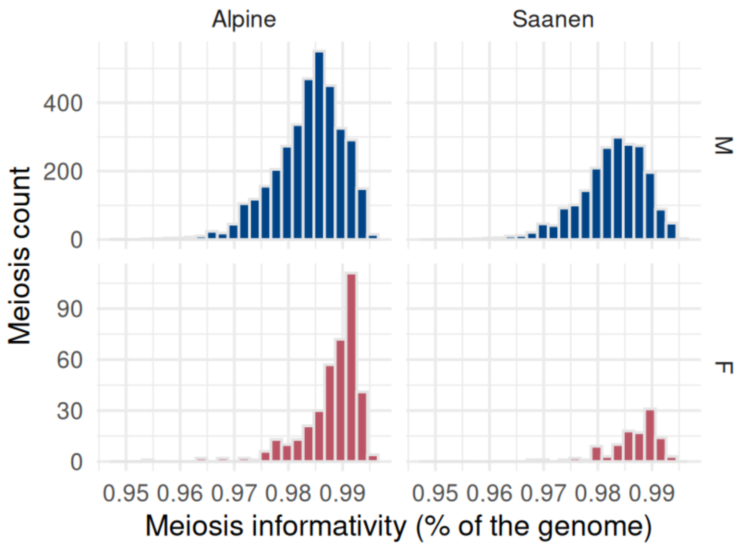

Figure S11: Distribution the proportion of the autosomal genome informative for crossovers in parents (M=males vs F=females).

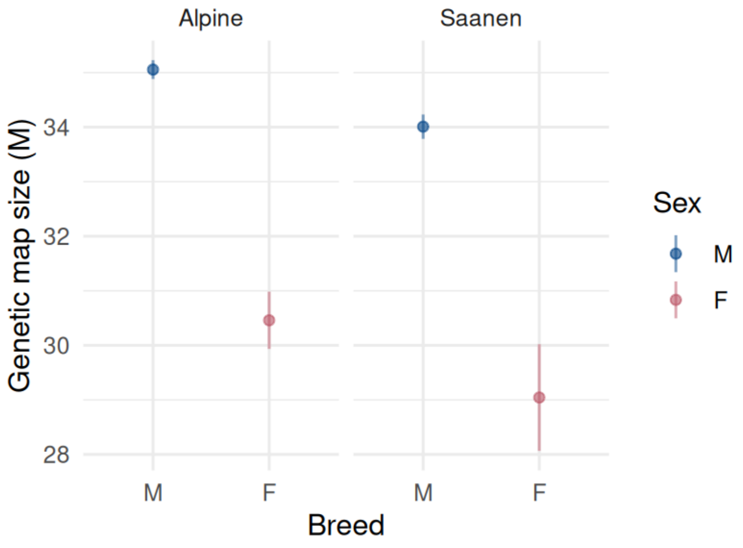

Figure S12: Comparison of recombination map size estimates between populations. Confidence interval = $\mu \pm 2 \times standard\ error$.

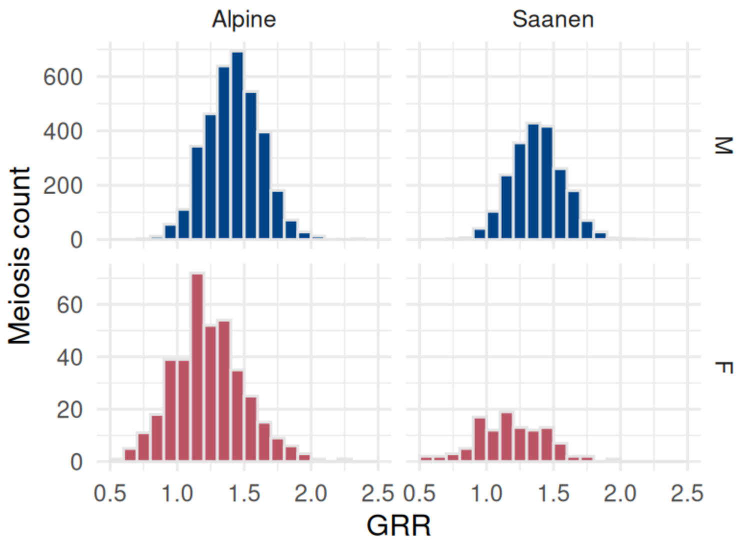

Figure S13: Distribution of genome-wide recombination rates (GRR) in meioses.

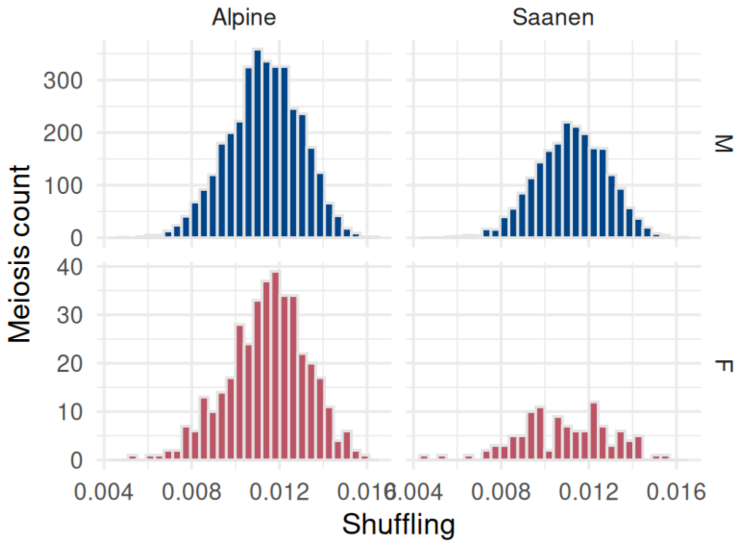

Figure S14: Distribution of intra-chromosomic shuffling in meioses.

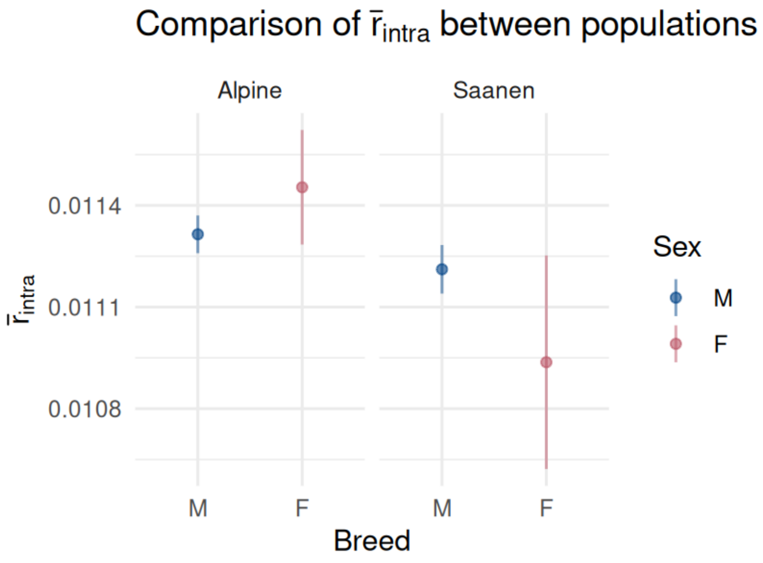

Figure S15: Comparison of intra-chromosomic shuffling estimates between populations. Confidence interval = $\mu \pm 2 \times standard\ error$.

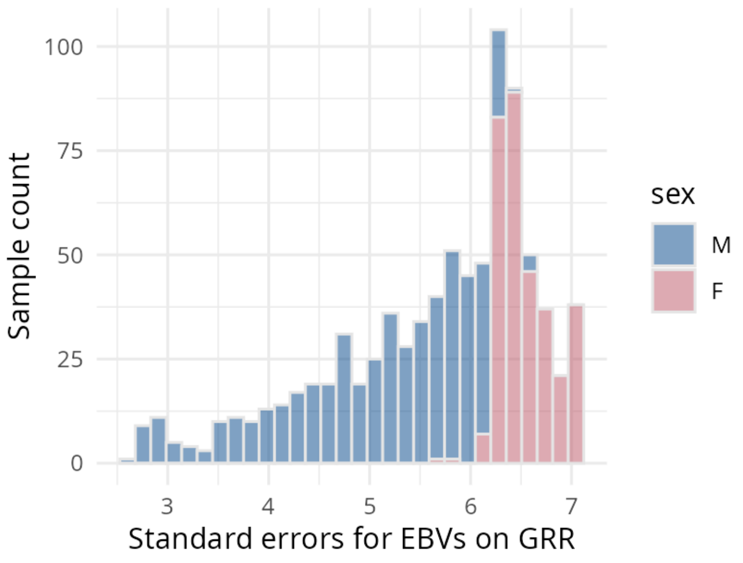

Figure S16: Distribution of standard errors for EBVs on GRR.

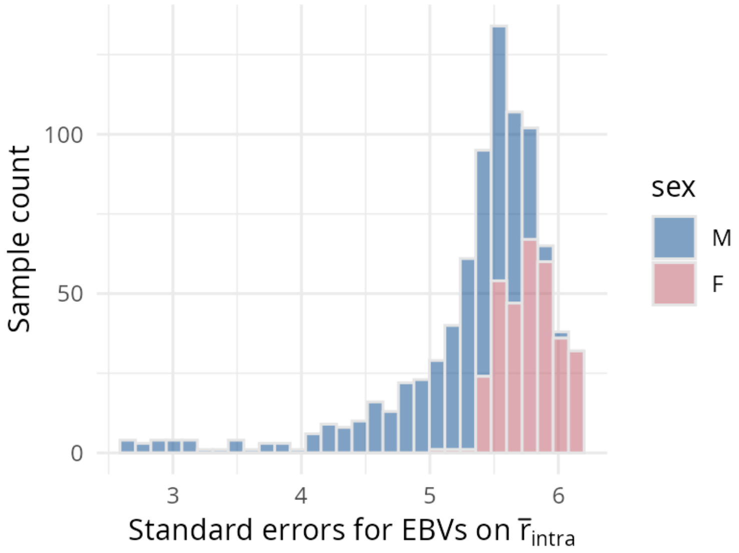

Figure S17: Distribution of standard errors for EBVs on $\bar{r}_{intra}$
