# Supplementary material for: Genome landscape and genetic architecture of recombination in domestic goats (Capra hircus)
Source: Genet Sel Evol. 2025 Oct 16;57:57. doi: 10.1186/s12711-025-01001-0 (PMC12532432; doi:10.1186/s12711-025-01001-0)
Supplement: Supplementary file 5 — Additional file 5: Figure S18. Estimated recombination rates for males and females on chromosome 1, at 1Mb resolution. Log ratio between male and female recombination rates were plotted in the bottom panel. Confidence interval of log ratio under null hypothesis appears in grey. Intervals found significant for sex differences are marked with a red dot on the bottom panel. Figure S19. Estimated recombination rates for males and females on chromosome 2, at 1Mb resolution. Log ratio between male and female recombination rates were plotted in the bottom panel. Confidence interval of log ratio under null hypothesis appears in grey. Intervals found significant for sex differences are marked with a red dot on the bottom panel. Figure S20. Estimated recombination rates for males and females on chromosome 3, at 1Mb resolution. Log ratio between male and female recombination rates were plotted in the bottom panel. Confidence interval of log ratio under null hypothesis appears in grey. Intervals found significant for sex differences are marked with a red dot on the bottom panel. Figure S21. Estimated recombination rates for males and females on chromosome 4, at 1Mb resolution. Log ratio between male and female recombination rates were plotted in the bottom panel. Confidence interval of log ratio under null hypothesis appears in grey. Intervals found significant for sex differences are marked with a red dot on the bottom panel. Figure S22. Estimated recombination rates for males and females on chromosome 5, at 1Mb resolution. Log ratio between male and female recombination rates were plotted in the bottom panel. Confidence interval of log ratio under null hypothesis appears in grey. Intervals found significant for sex differences are marked with a red dot on the bottom panel. Figure S23. Estimated recombination rates for males and females on chromosome 6, at 1Mb resolution. Log ratio between male and female recombination rates were plotted in the bottom panel. Confidence interva [file 12711_2025_1001_MOESM5_ESM.pdf]

Chromosome-specific recombination maps

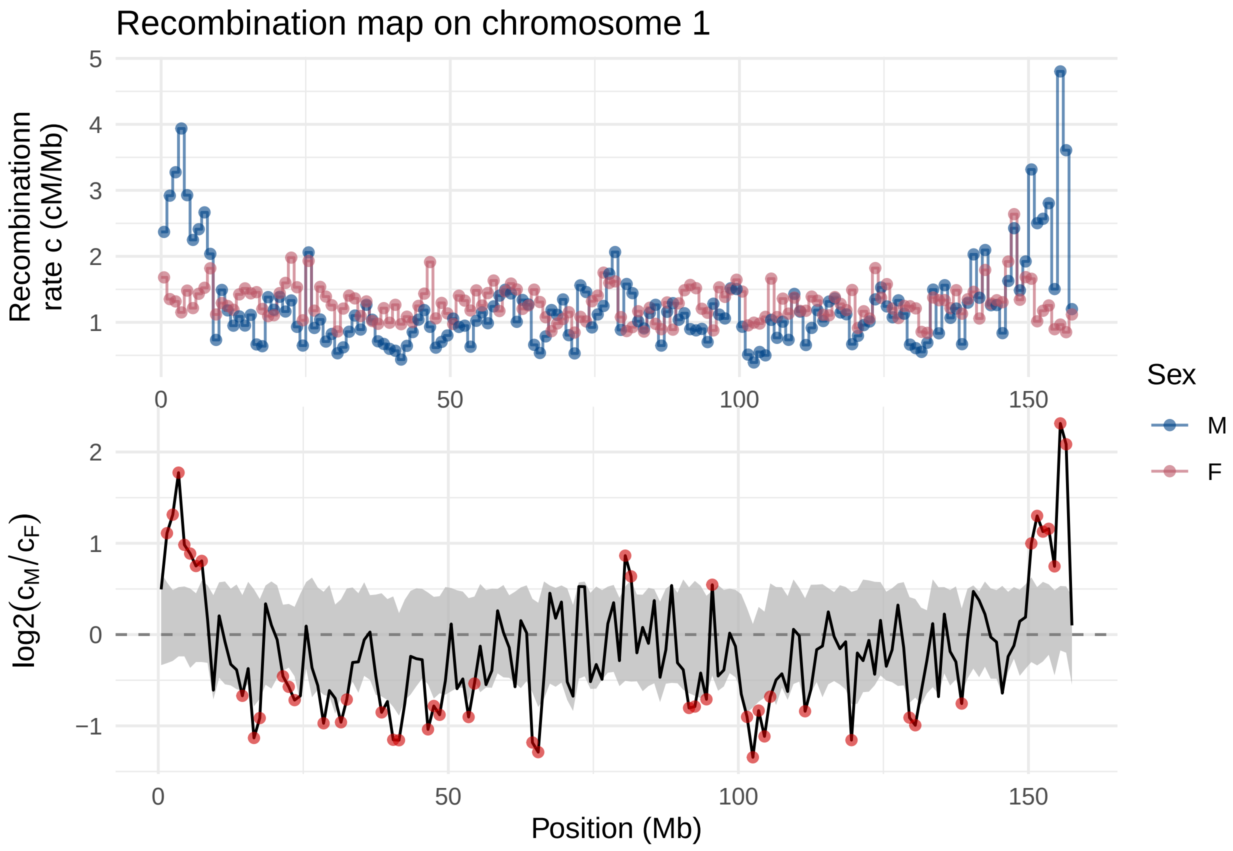

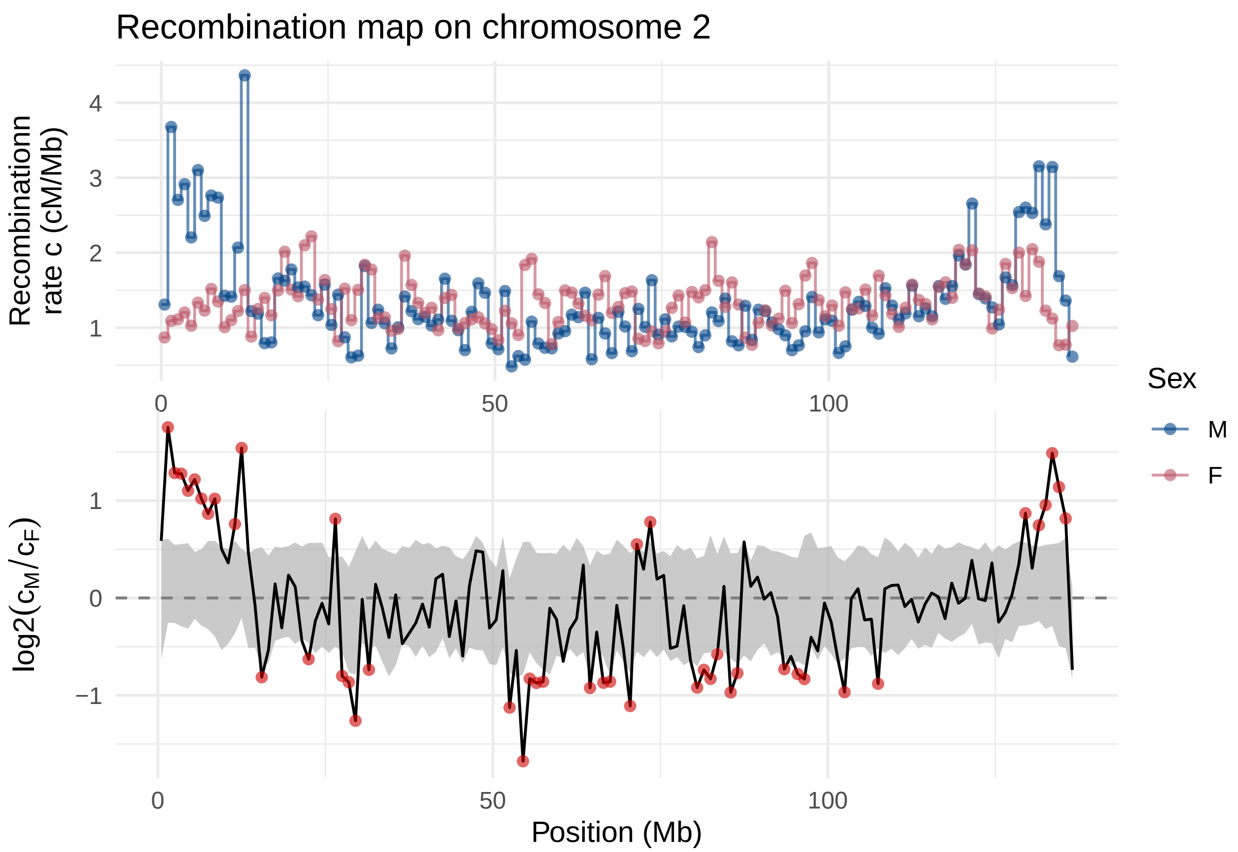
Recombination map on chromosome 2
Recombinationn rate c (cM/Mb)
4
3
2
1
0
50
100
Sex
M
F
log2(cM/cF)
1
0
−1
0
50
100
Position (Mb)

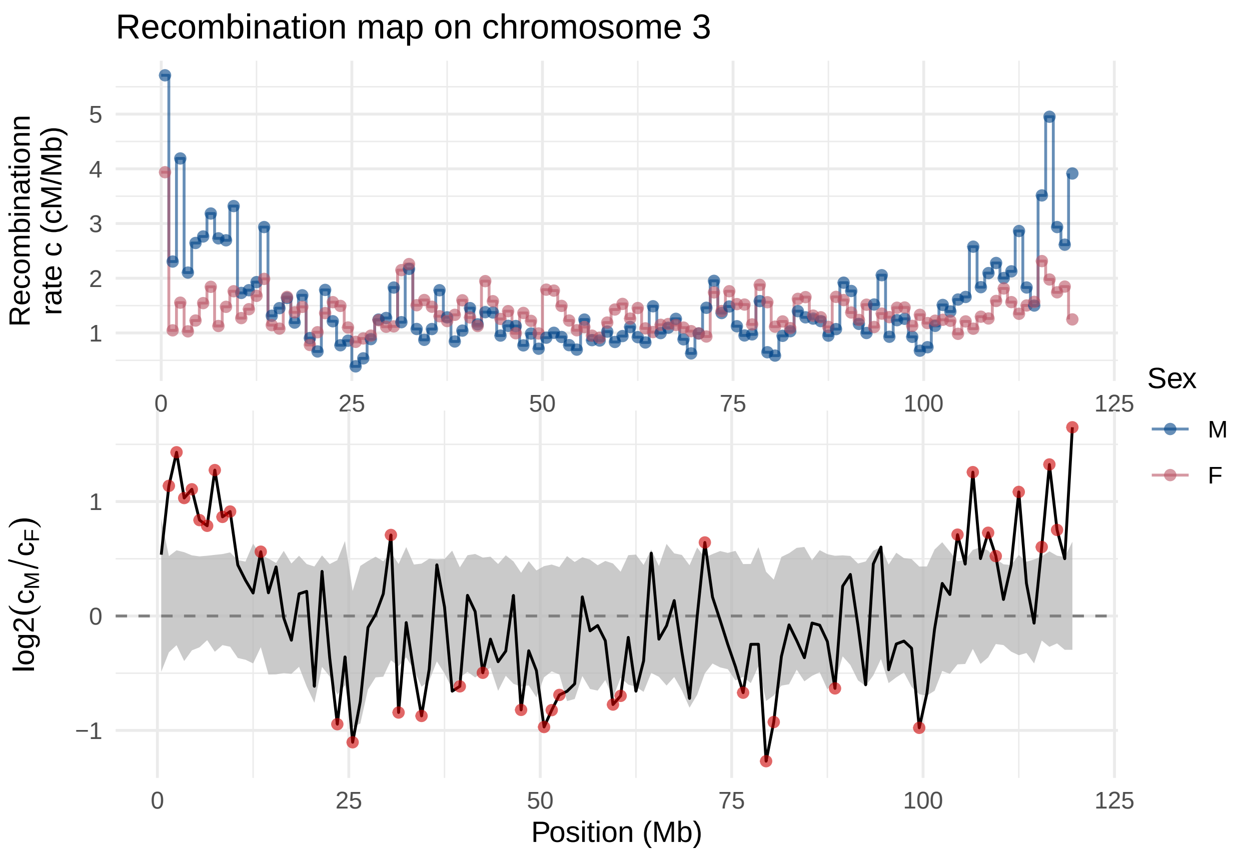
Recombination map on chromosome 3
Recombinationn rate c (cM/Mb)
5
4
3
2
1
0
25
50
75
100
125
Sex
M
F
$\log2(c_M/c_F)$
1
0
−1
Position (Mb)

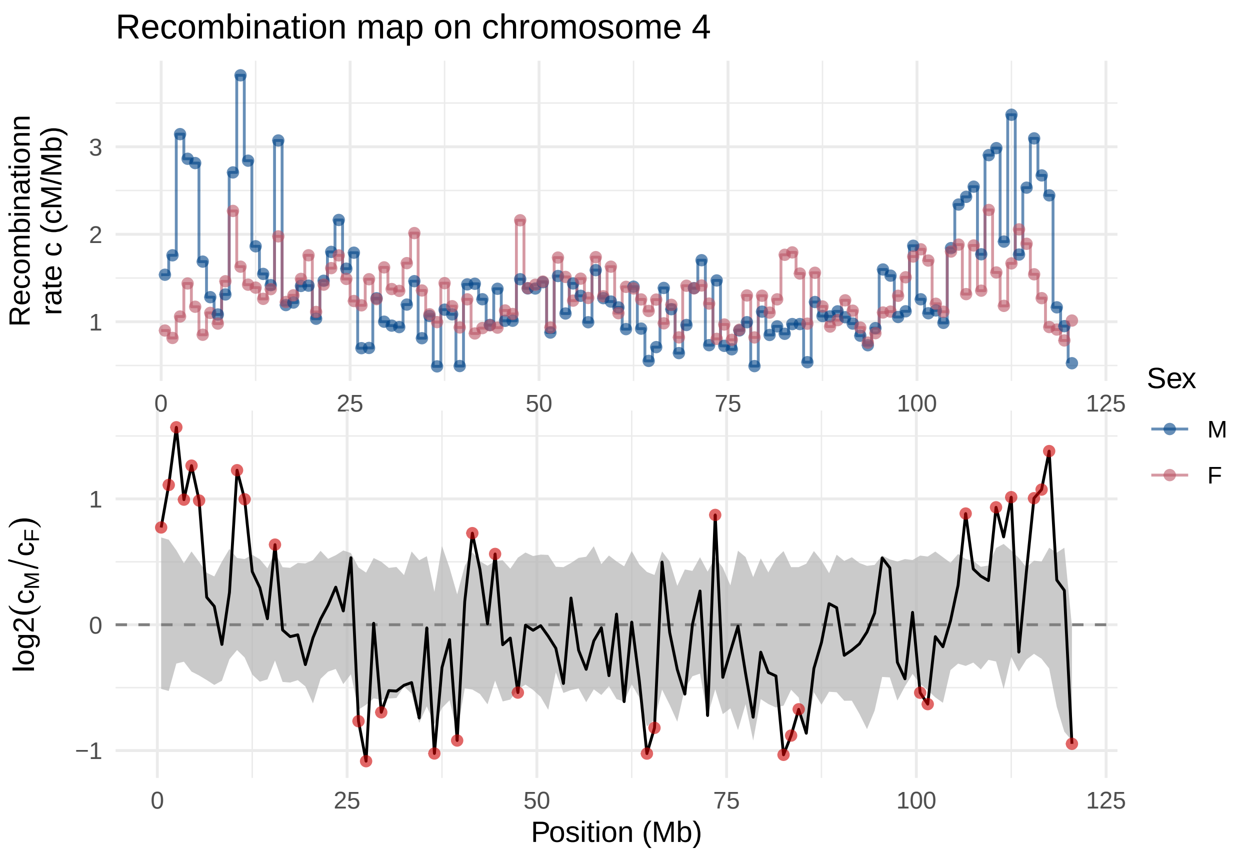

Recombination map on chromosome 4
Recombinationn rate c (cM/Mb)
log2(c_M/c_F)
Position (Mb)
Sex
M
F
0
25
50
75
100
125
1
2
3
0
−1

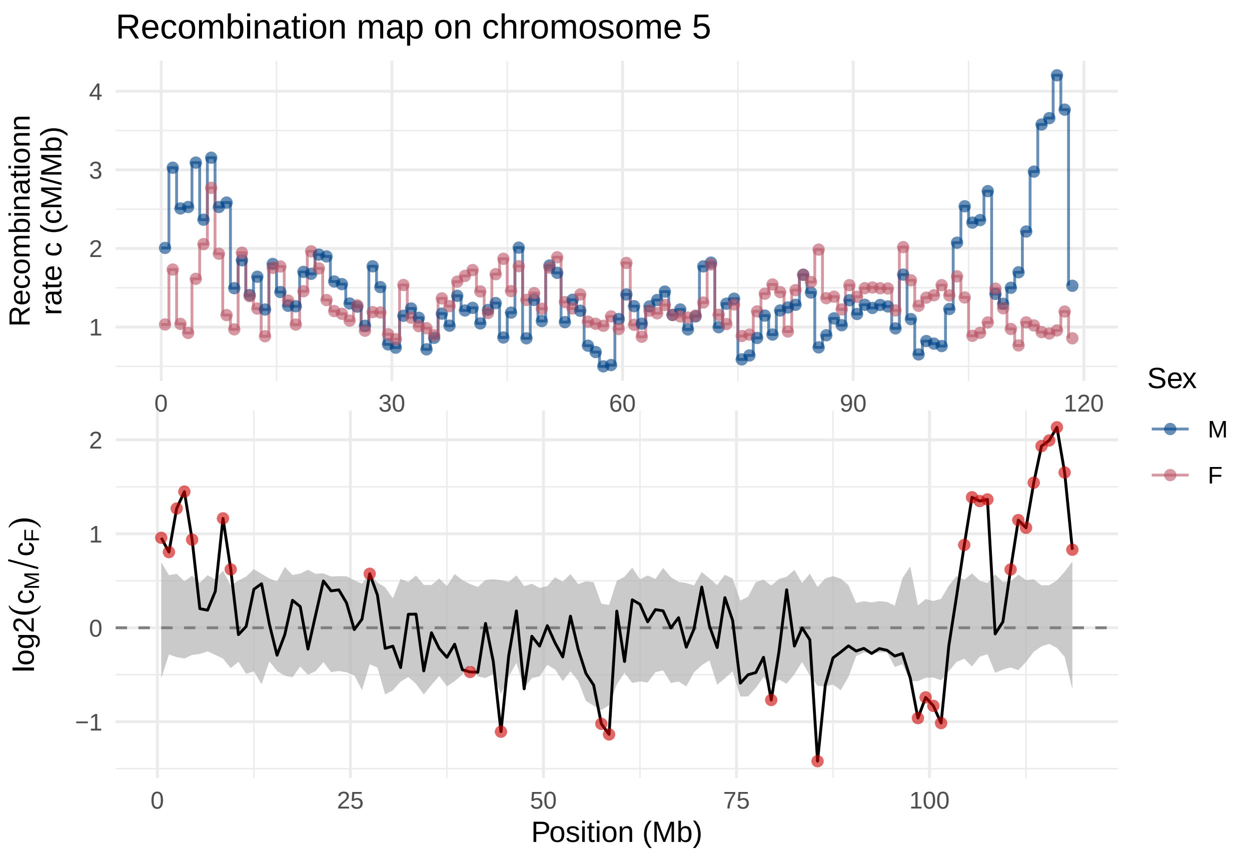
Recombination map on chromosome 5
Recombinationn
rate c (cM/Mb)
4
3
2
1
0
30
60
90
120
Sex
M
F
log2(cM/cF)
2
1
0
−1
0
25
50
75
100
Position (Mb)

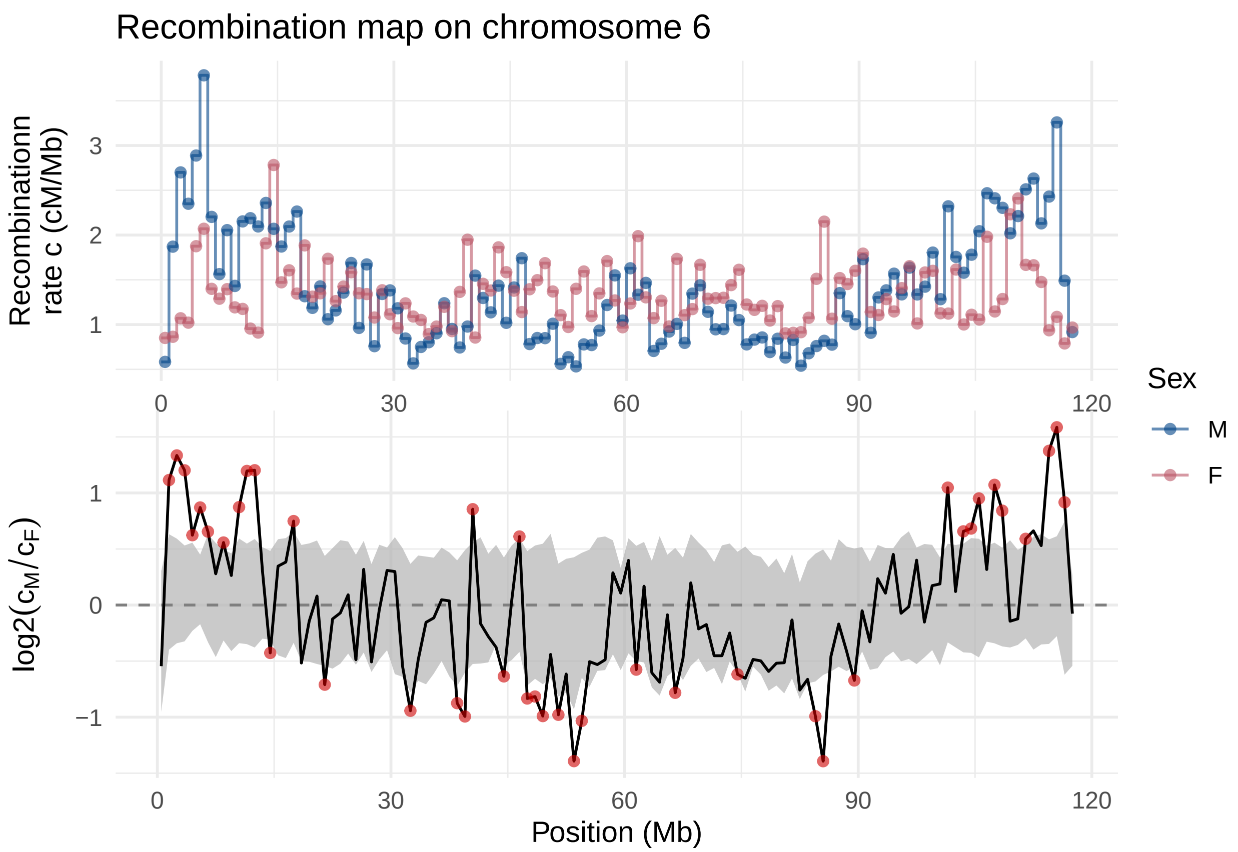

Recombination map on chromosome 6
Recombinationn rate c (cM/Mb)
3
2
1
0
30
60
90
120
Sex
M
F
log2(c_M/c_F)
1
0
−1
0
30
60
90
120
Position (Mb)

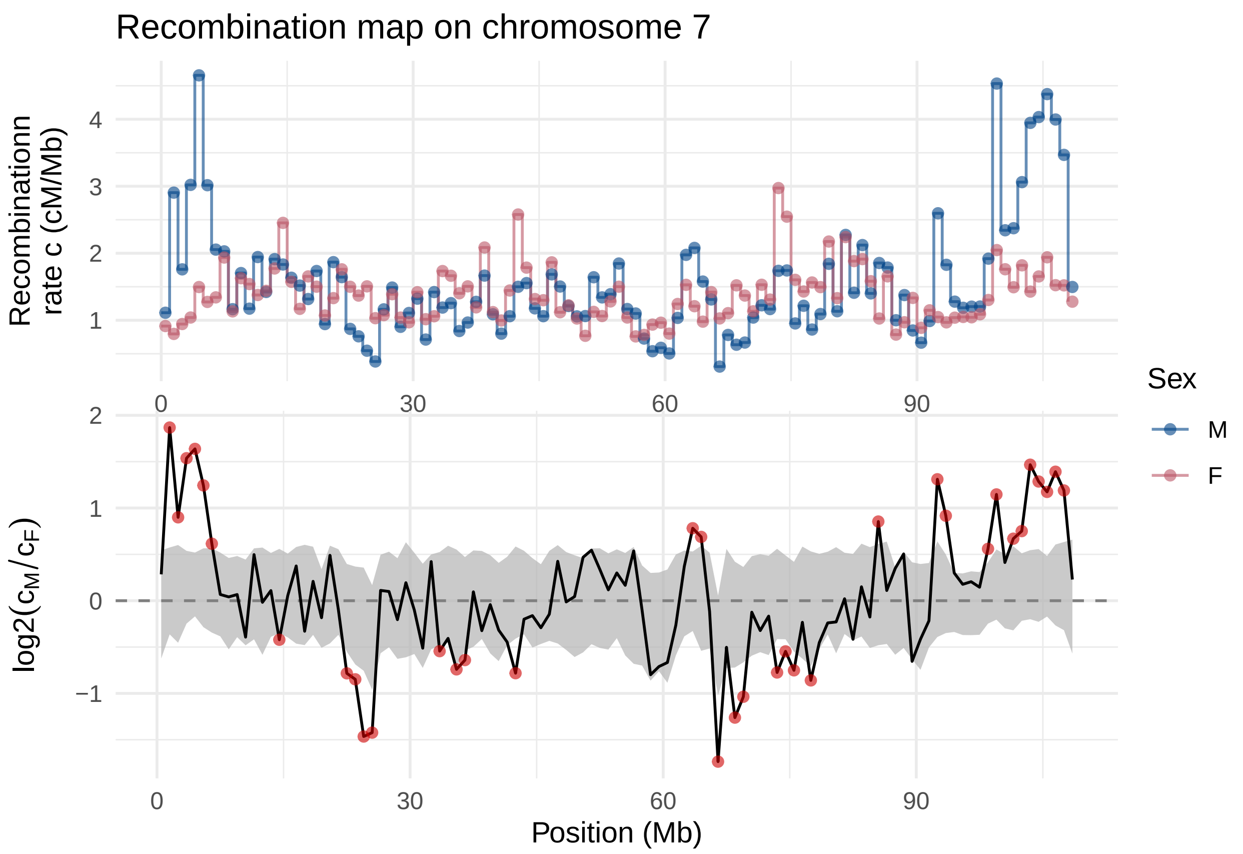

Recombination map on chromosome 7
Recombinationn rate c (cM/Mb)
4
3
2
1
0
30
60
90
Sex
M
F
log2(c_M/c_F)
2
1
0
−1
0
30
60
90
Position (Mb)

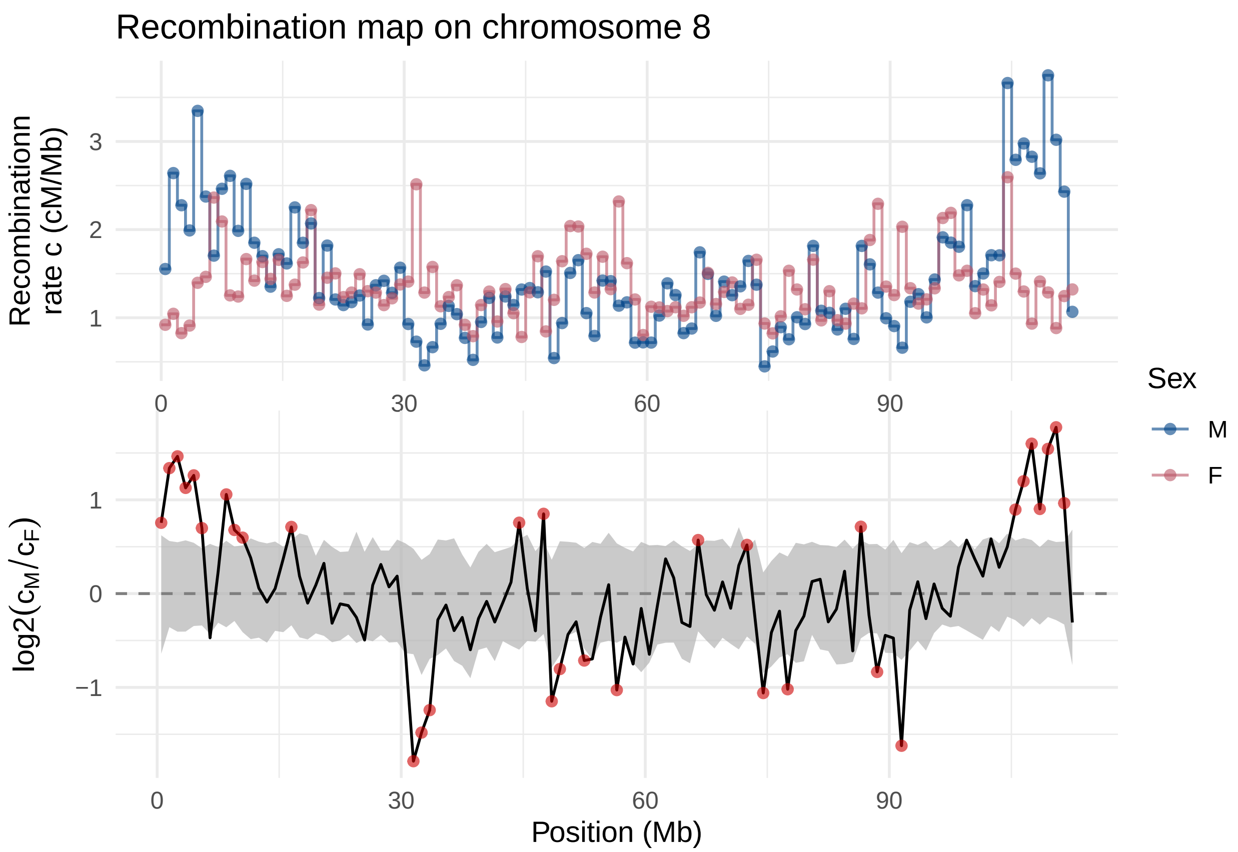

Recombination map on chromosome 8
Recombinationn rate c (cM/Mb)
3
2
1
0
30
60
90
Sex
M
F
log2(c_M/c_F)
1
0
−1
0
30
60
90
Position (Mb)

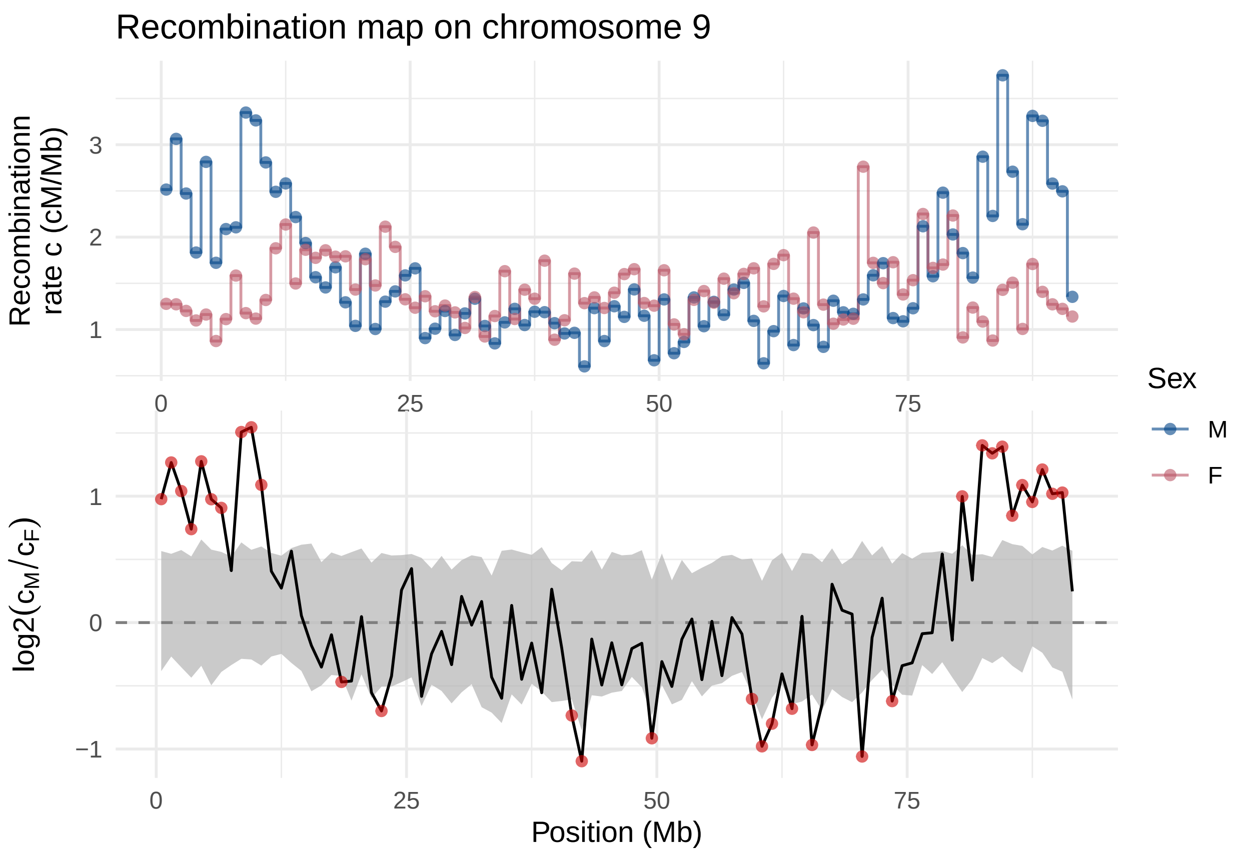

Recombination map on chromosome 9
Recombinationn rate c (cM/Mb)
3
2
1
0
25
50
75
Sex
M
F
log2(cM/cF)
1
0
−1
0
25
50
75
Position (Mb)

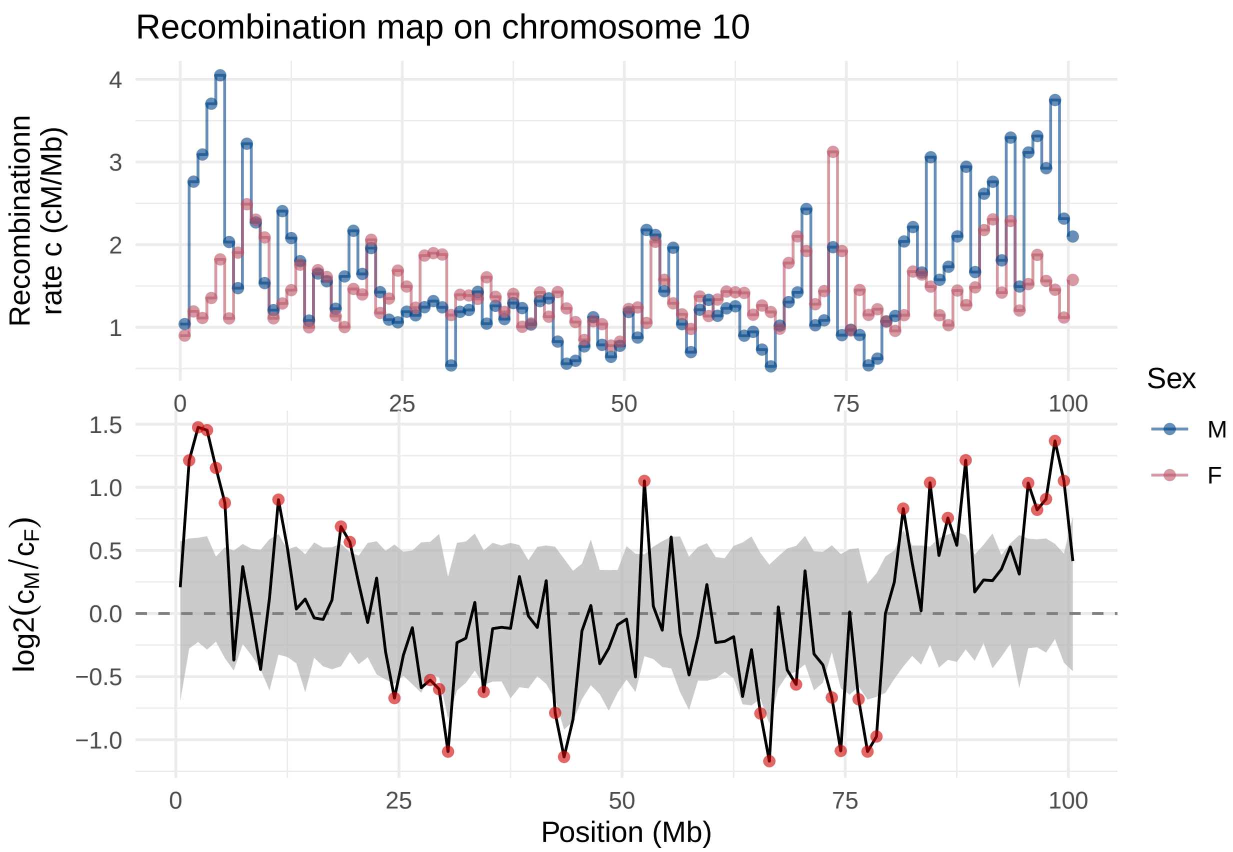
Recombination map on chromosome 10
Recombinationn rate c (cM/Mb)
Sex
M
F
log2(cM/cF)
Position (Mb)
0
25
50
75
100
1
2
3
4
1.5
1.0
0.5
0.0
−0.5
−1.0

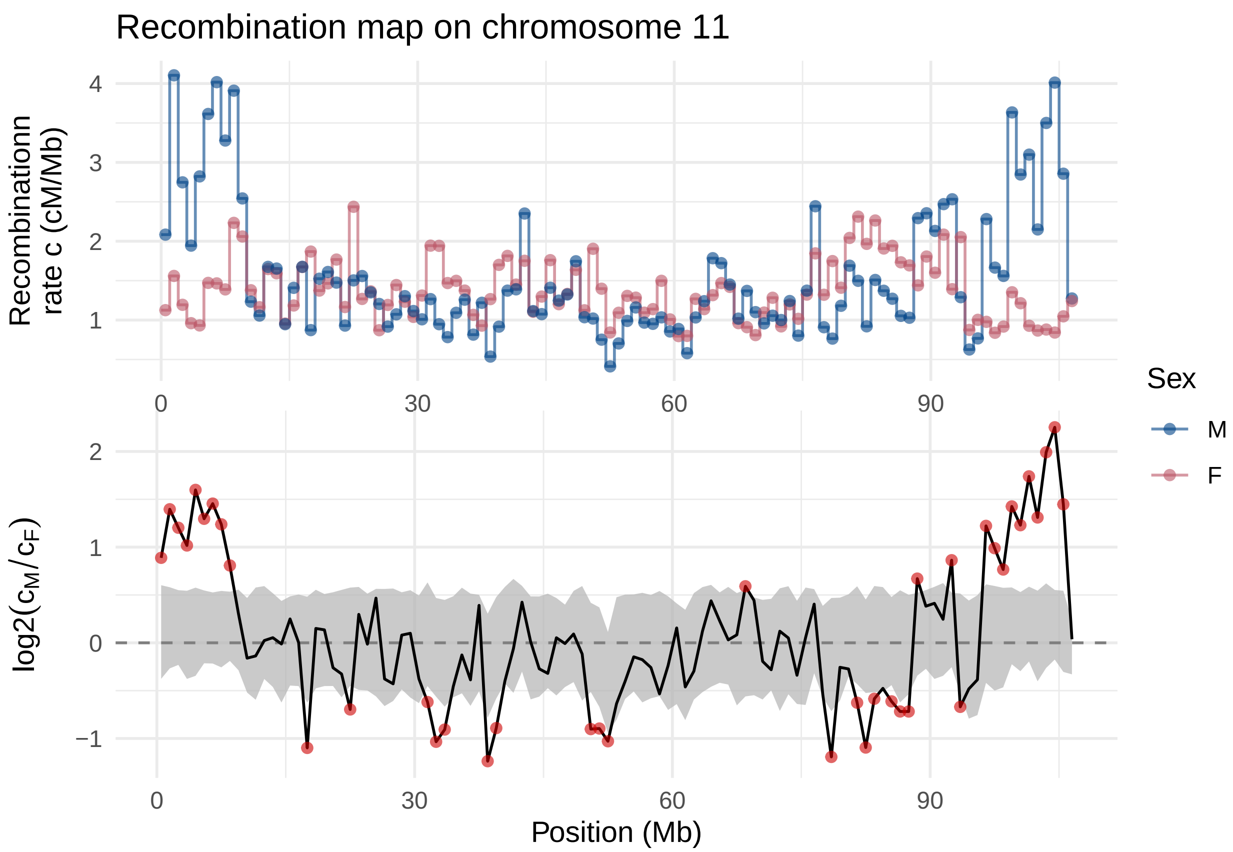

Recombination map on chromosome 11
Recombinationn rate c (cM/Mb)
log2(cM/cF)
Position (Mb)
Sex
M
F

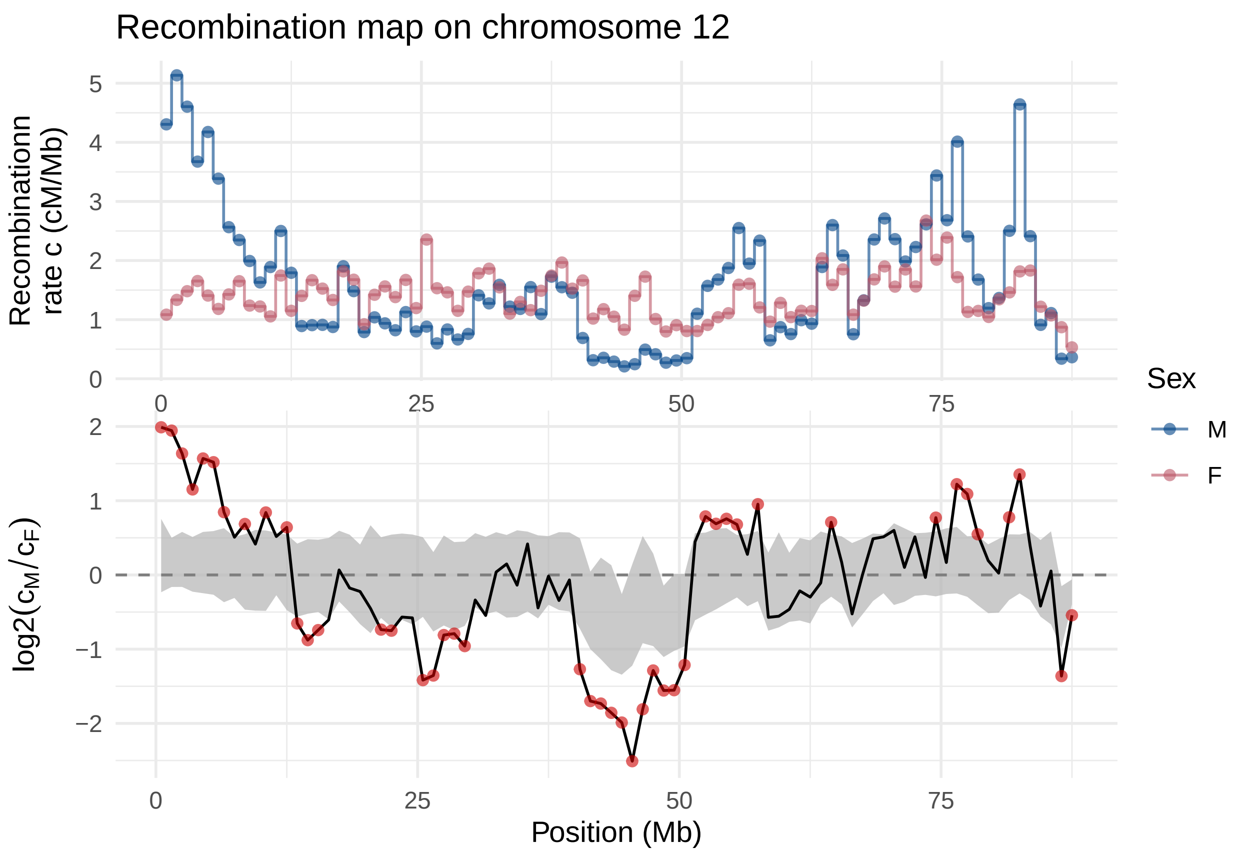
Recombination map on chromosome 12
Recombinationn rate c (cM/Mb)
log2(cM/cF)
Position (Mb)
Sex
M
F
0
25
50
75
5
4
3
2
1
0
−1
−2

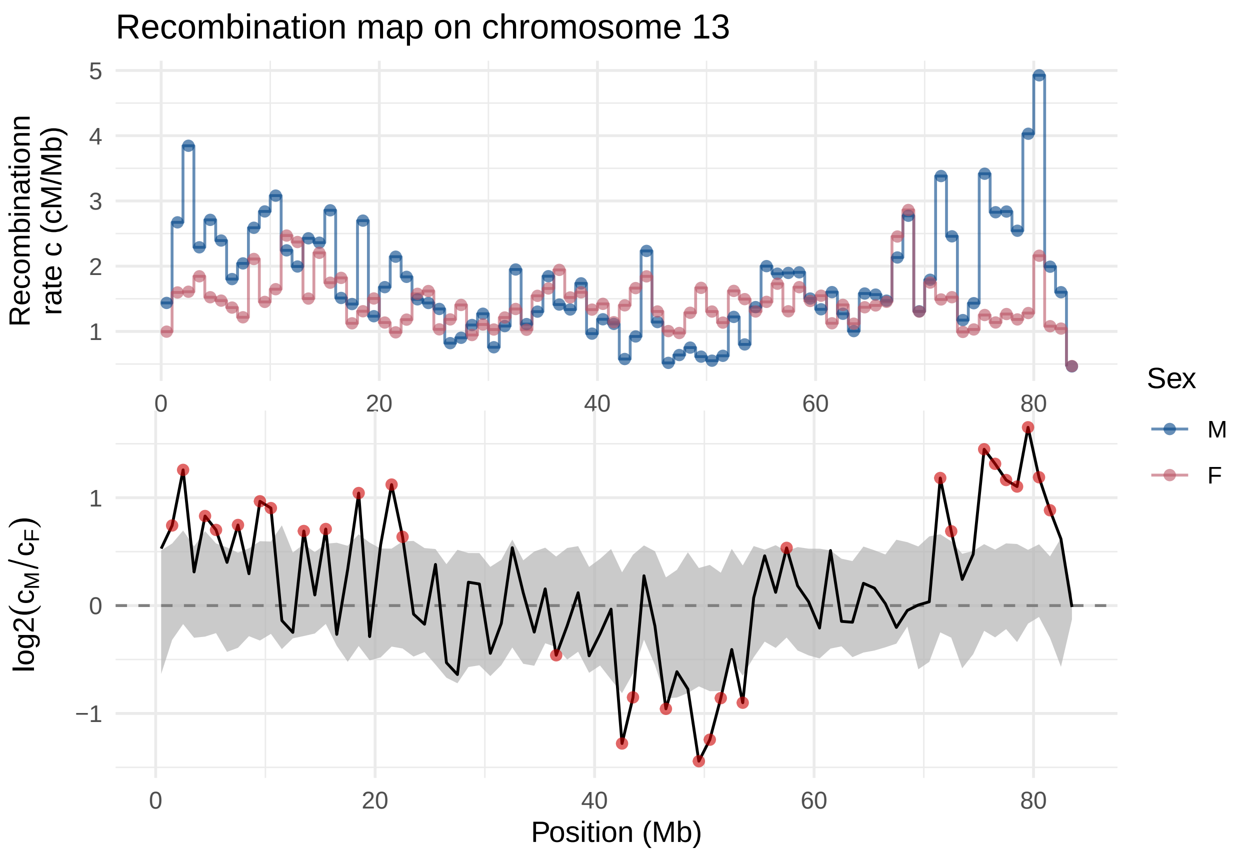

Recombination map on chromosome 13
Recombinationn rate c (cM/Mb)
5
4
3
2
1
0
20
40
60
80
log2(c_M/c_F)
1
0
−1
0
20
40
60
80
Position (Mb)
Sex
M
F

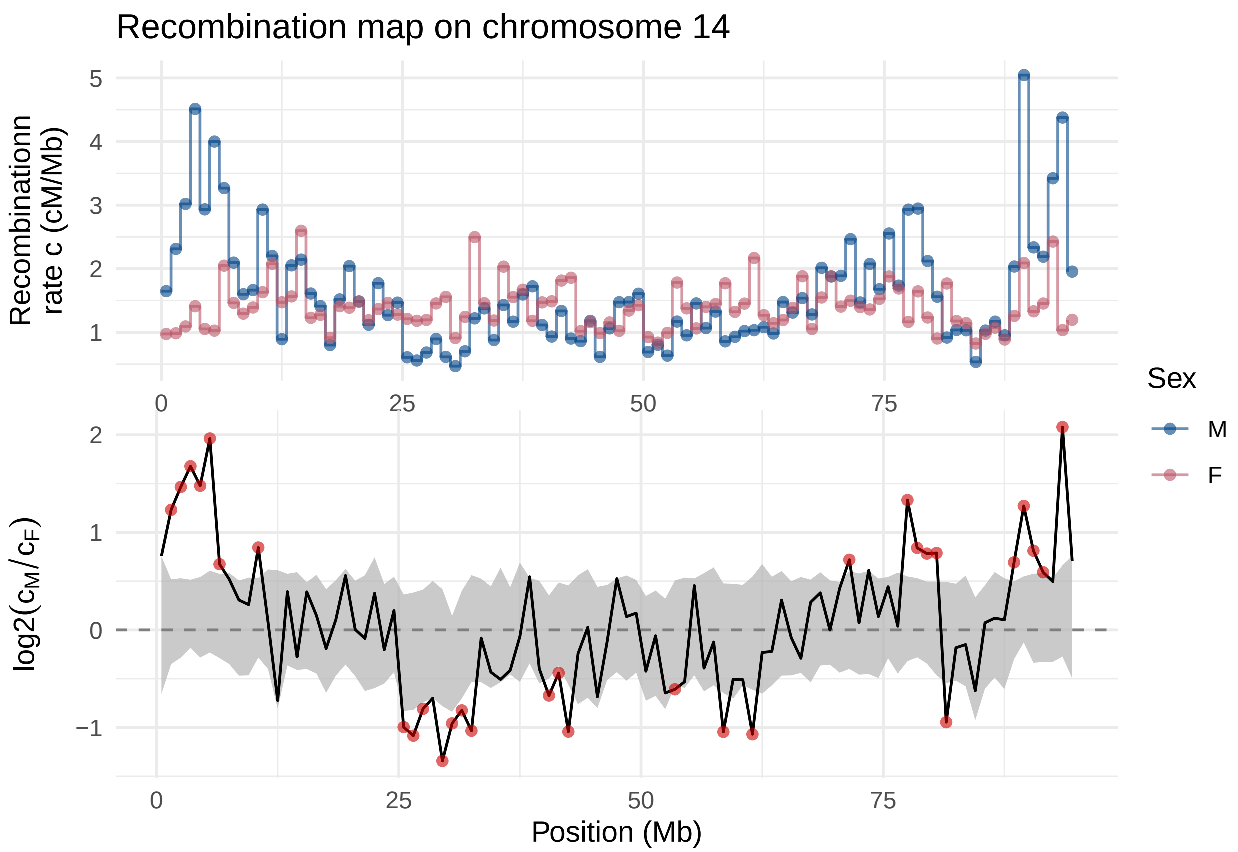
Recombination map on chromosome 14
Recombinationn rate c (cM/Mb)
5
4
3
2
1
0
25
50
75
Sex
M
F
log2(c_M/c_F)
2
1
0
−1
0
25
50
75
Position (Mb)

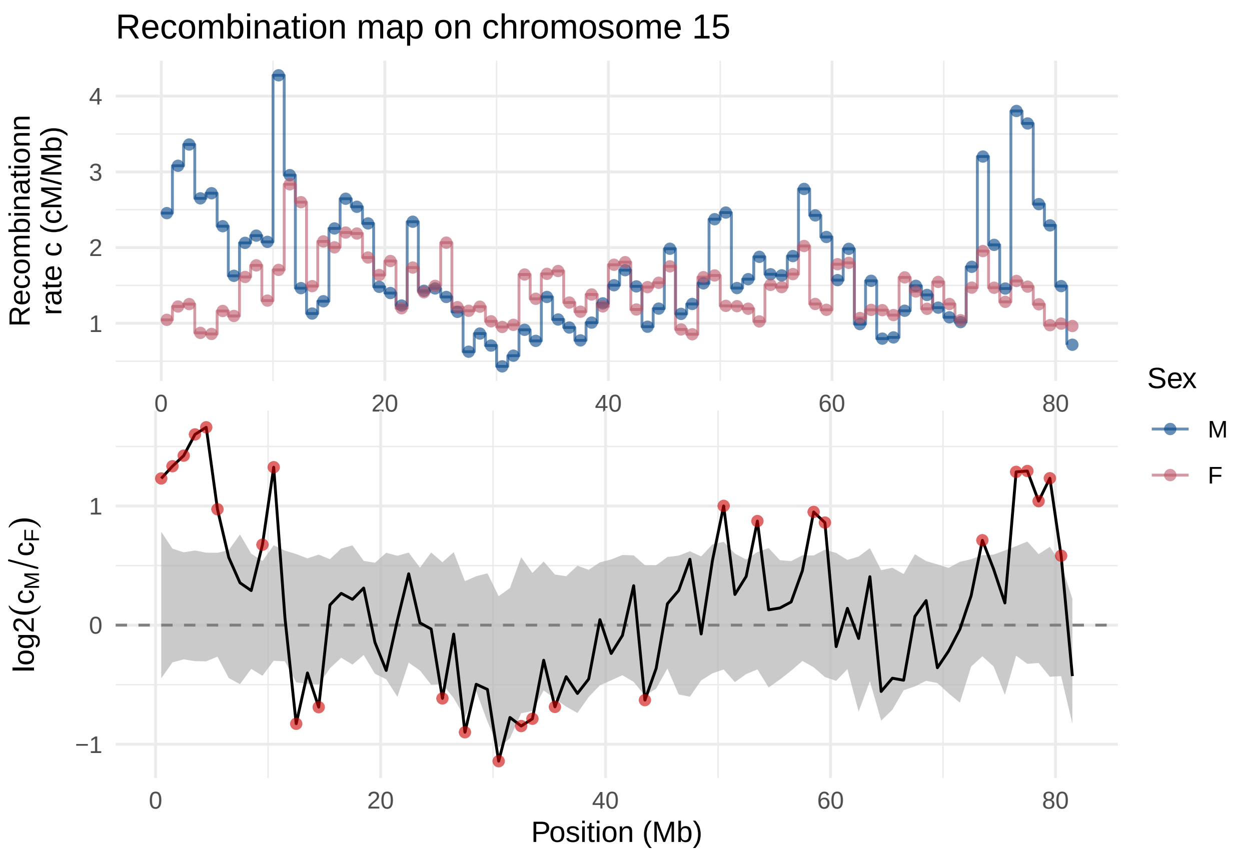
Recombination map on chromosome 15
Recombinationn rate c (cM/Mb)
4
3
2
1
0
20
40
60
80
Sex
M
F
log2(cM/cF)
1
0
−1
Position (Mb)

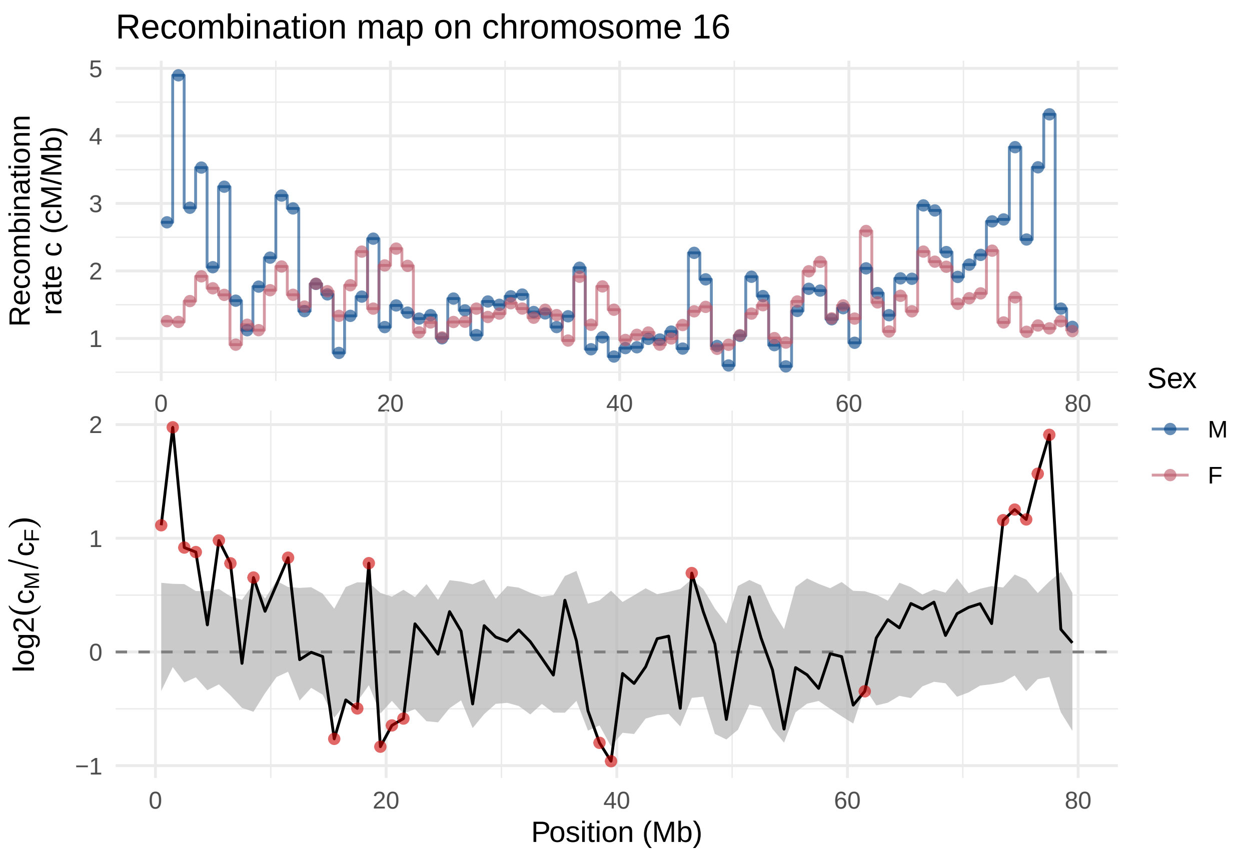
Recombination map on chromosome 16
Recombinationn
rate c (cM/Mb)
5
4
3
2
1
0
20
40
60
80
Sex
M
F
log2(cM/cF)
2
1
0
−1
0
20
40
60
80
Position (Mb)

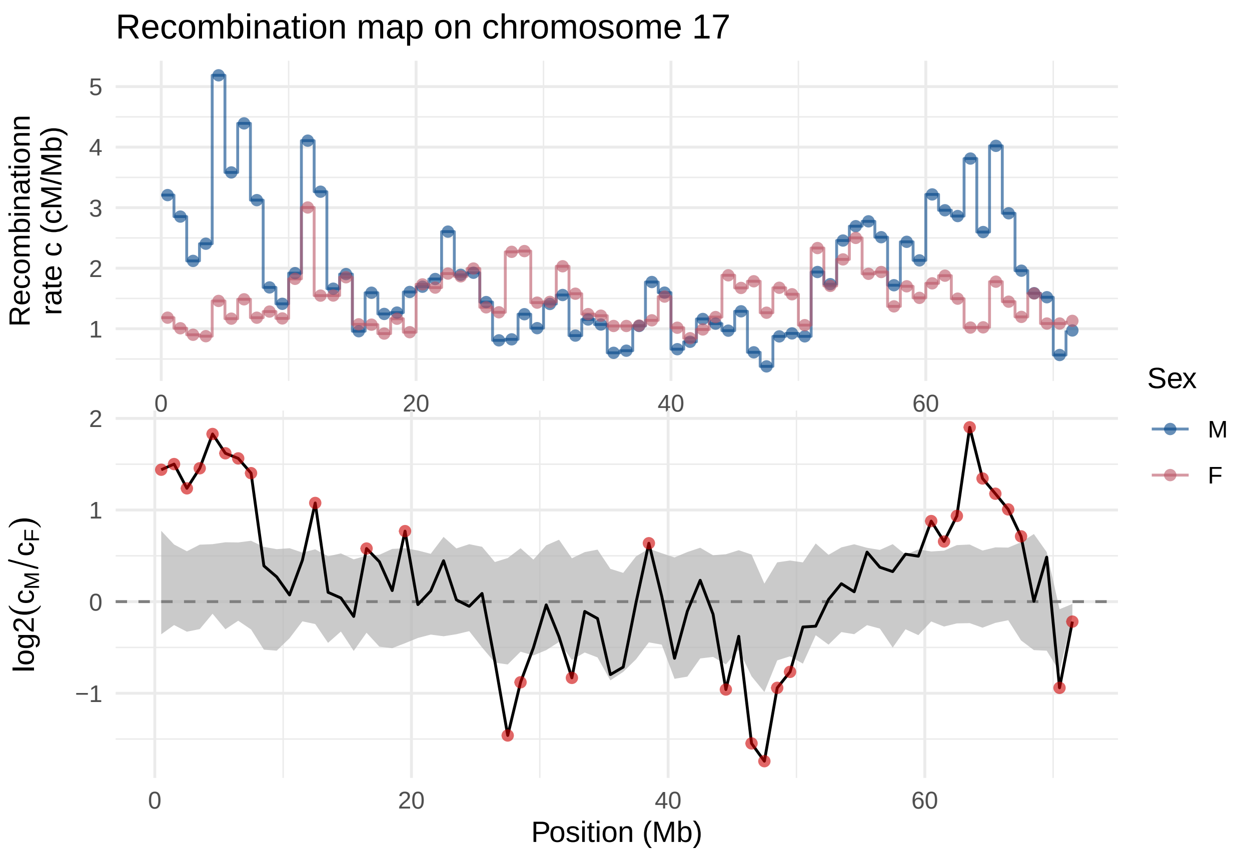

Recombination map on chromosome 17
Recombinationn
rate c (cM/Mb)
5
4
3
2
1
0
20
40
60
Sex
M
F
2
1
0
−1
log2(cM/cF)
0
20
40
60
Position (Mb)

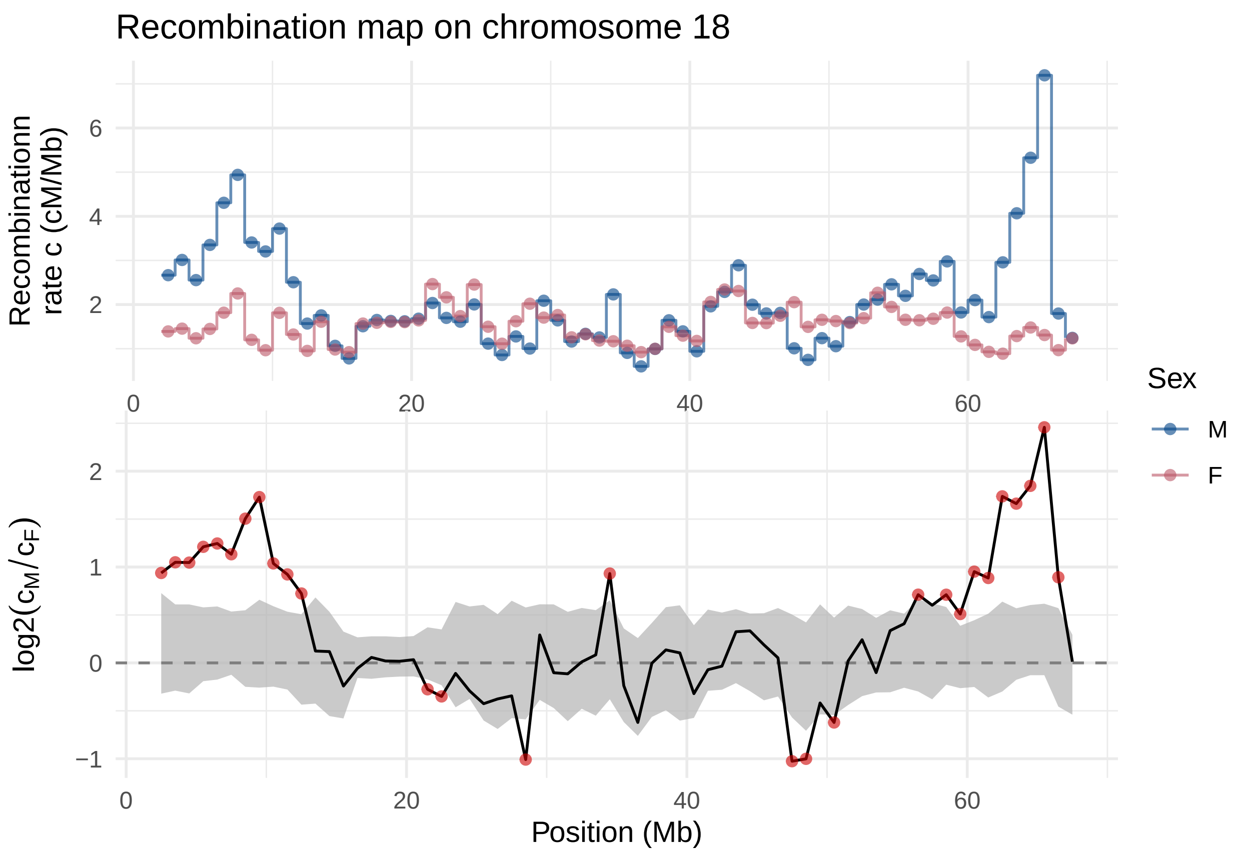
Recombination map on chromosome 18
Recombinationn rate c (cM/Mb)
log2(cM/cF)
Position (Mb)
Sex
M
F
0
20
40
60
2
4
6
−1
0
1
2

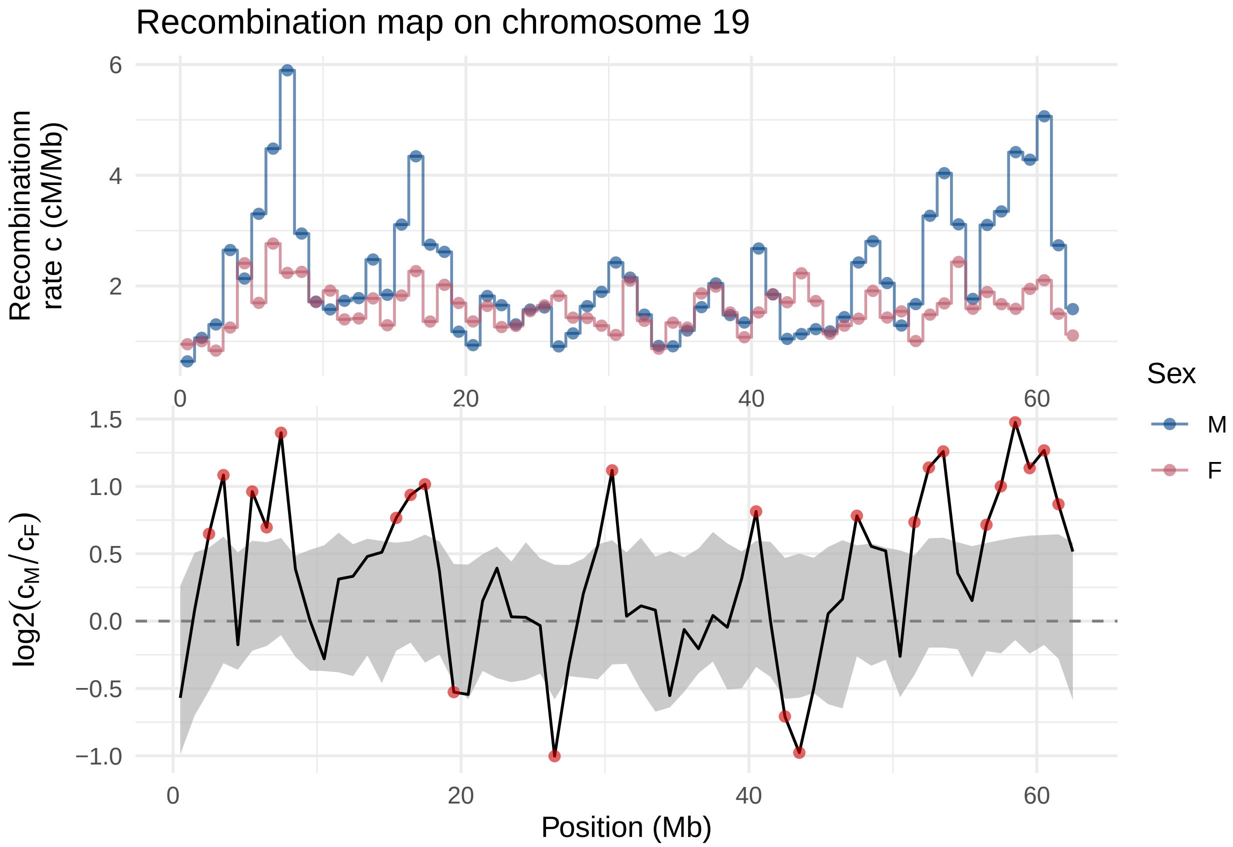
Recombination map on chromosome 19
Recombinationn rate c (cM/Mb)
log2(cM/cF)
Position (Mb)
Sex
M
F
6
4
2
0
20
40
60
1.5
1.0
0.5
0.0
−0.5
−1.0

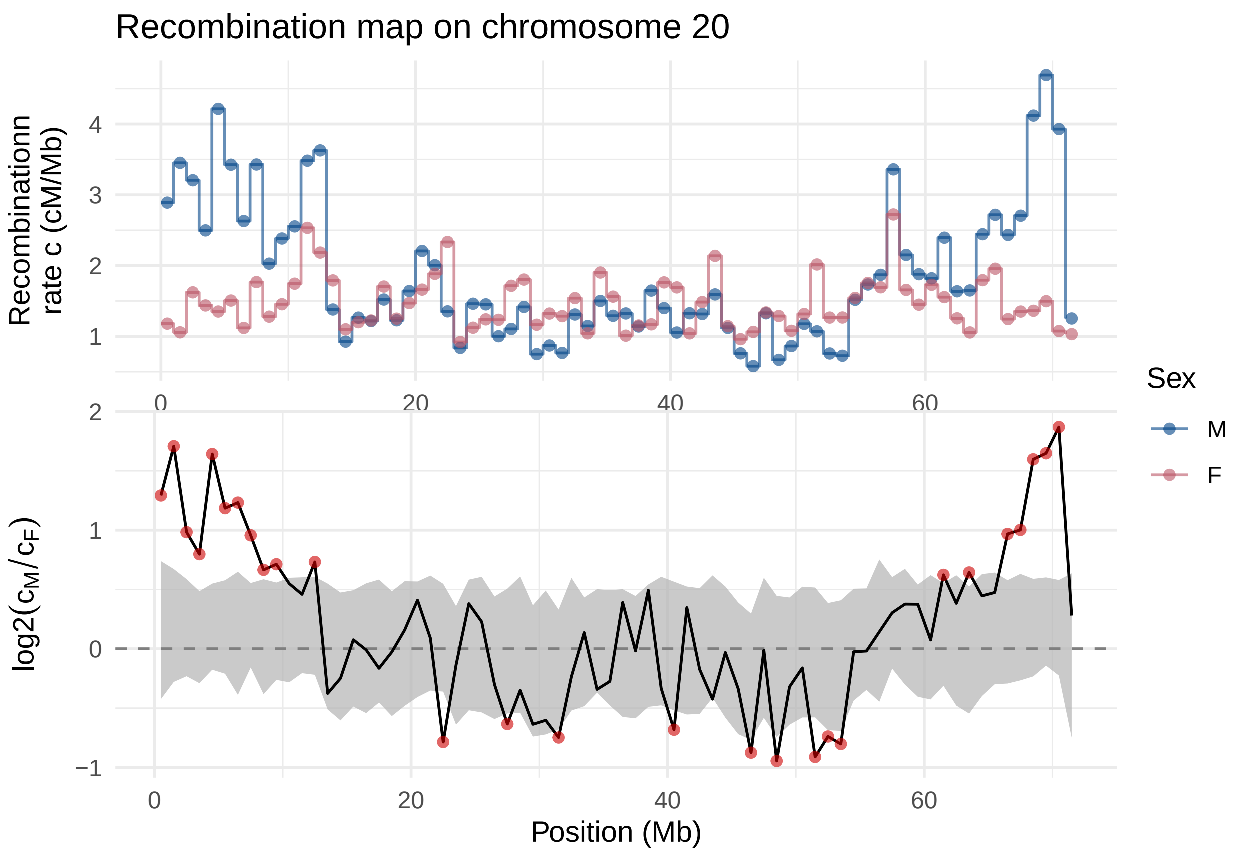
Recombination map on chromosome 20
Recombinationn rate c (cM/Mb)
4
3
2
1
0
20
40
60
Sex
M
F
log2(cM/cF)
2
1
0
−1
0
20
40
60
Position (Mb)

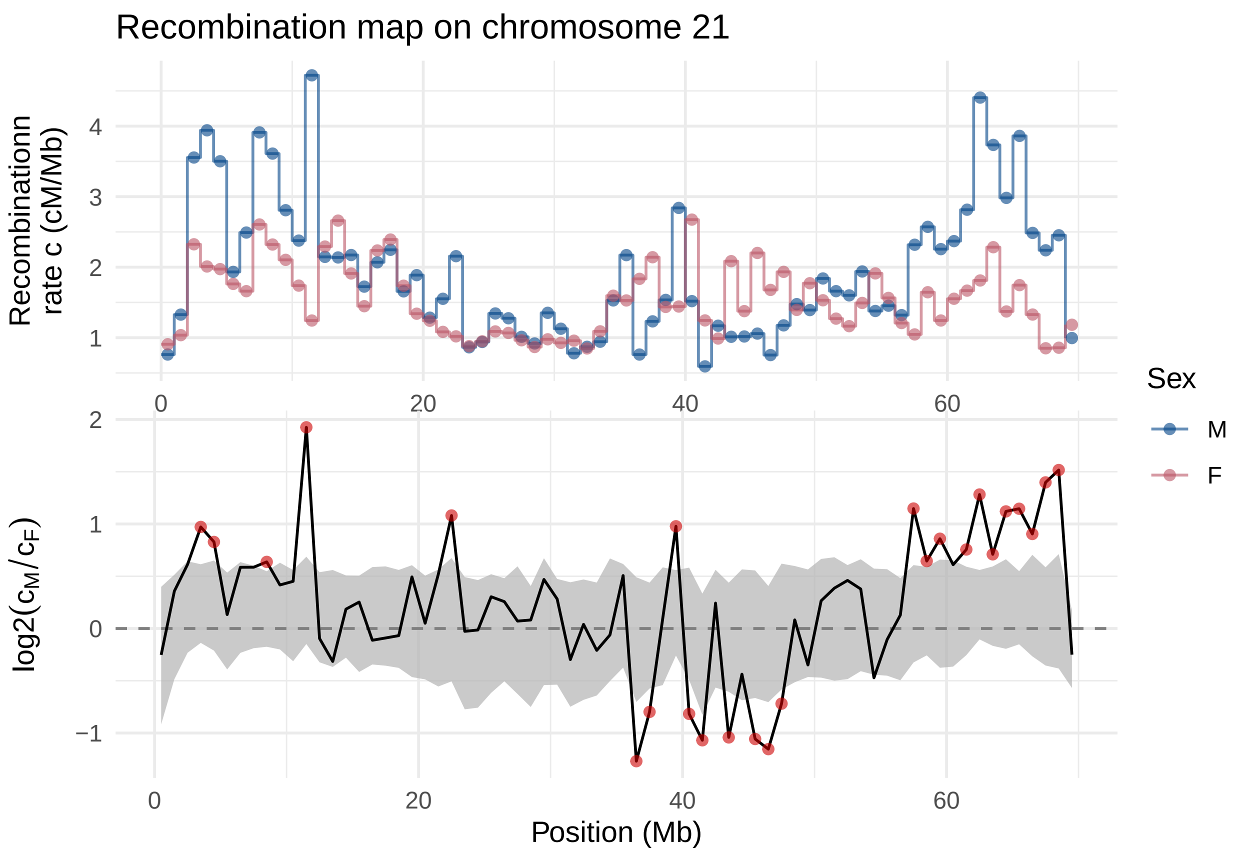

Recombination map on chromosome 21
Recombinationn rate c (cM/Mb)
log2(cM/cF)
Position (Mb)
Sex
M
F

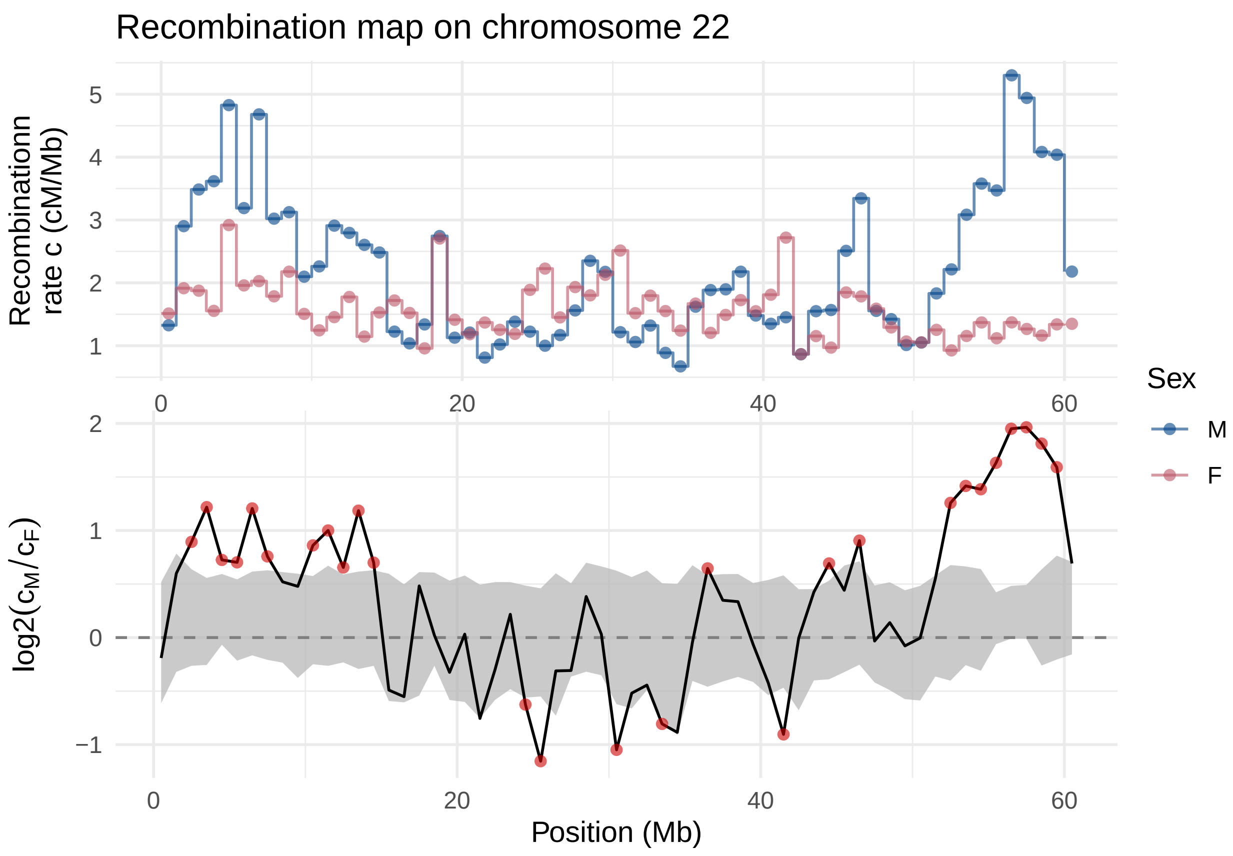

Recombination map on chromosome 22
Recombinationn rate c (cM/Mb)
5
4
3
2
1
0
20
40
60
Sex
M
F
log2(cM/cF)
2
1
0
−1
0
20
40
60
Position (Mb)

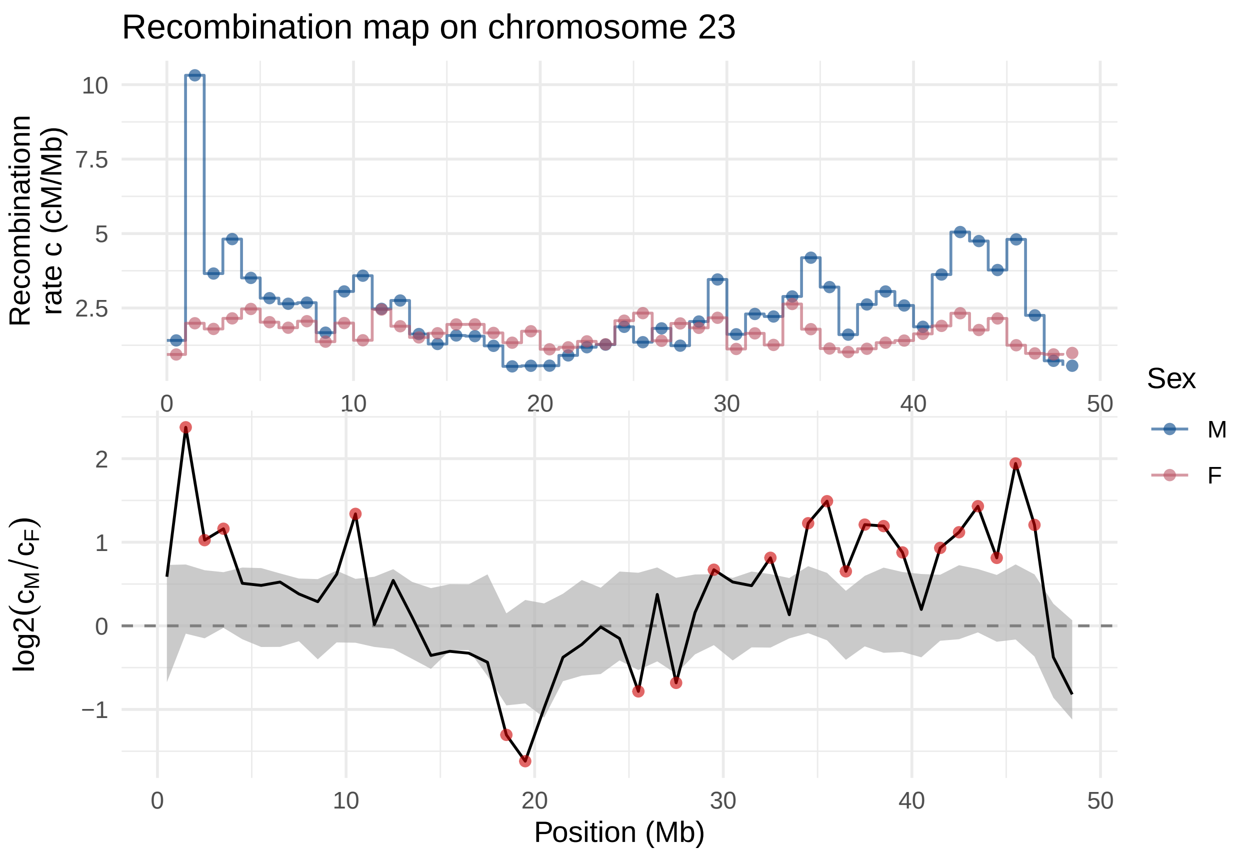
Recombination map on chromosome 23
Recombinationn rate c (cM/Mb)
10
7.5
5
2.5
0
10
20
30
40
50
Sex
M
F
log2(c_M/c_F)
2
1
0
−1
Position (Mb)

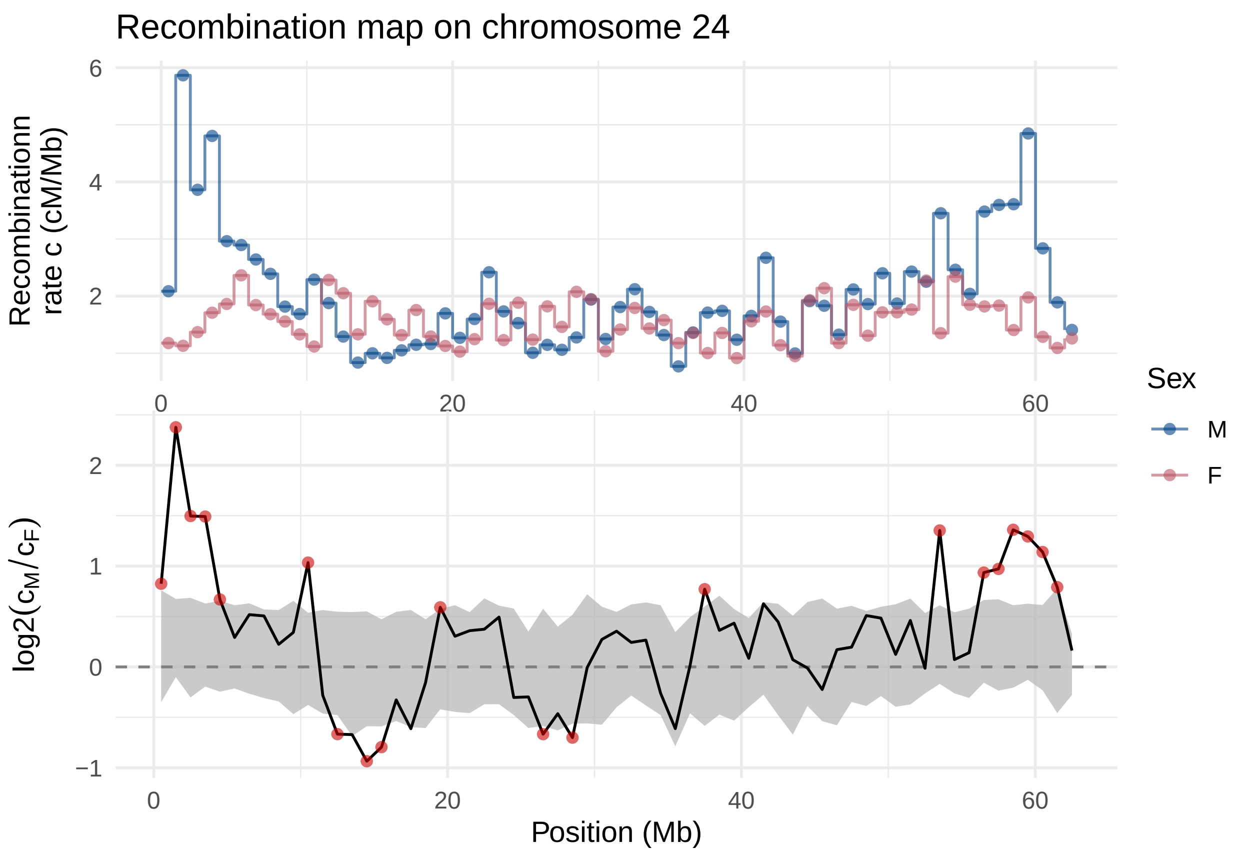
Recombination map on chromosome 24
Recombinationn rate c (cM/Mb)
6
4
2
0
20
40
60
Sex
M
F
log2(c_M/c_F)
2
1
0
−1
0
20
40
60
Position (Mb)

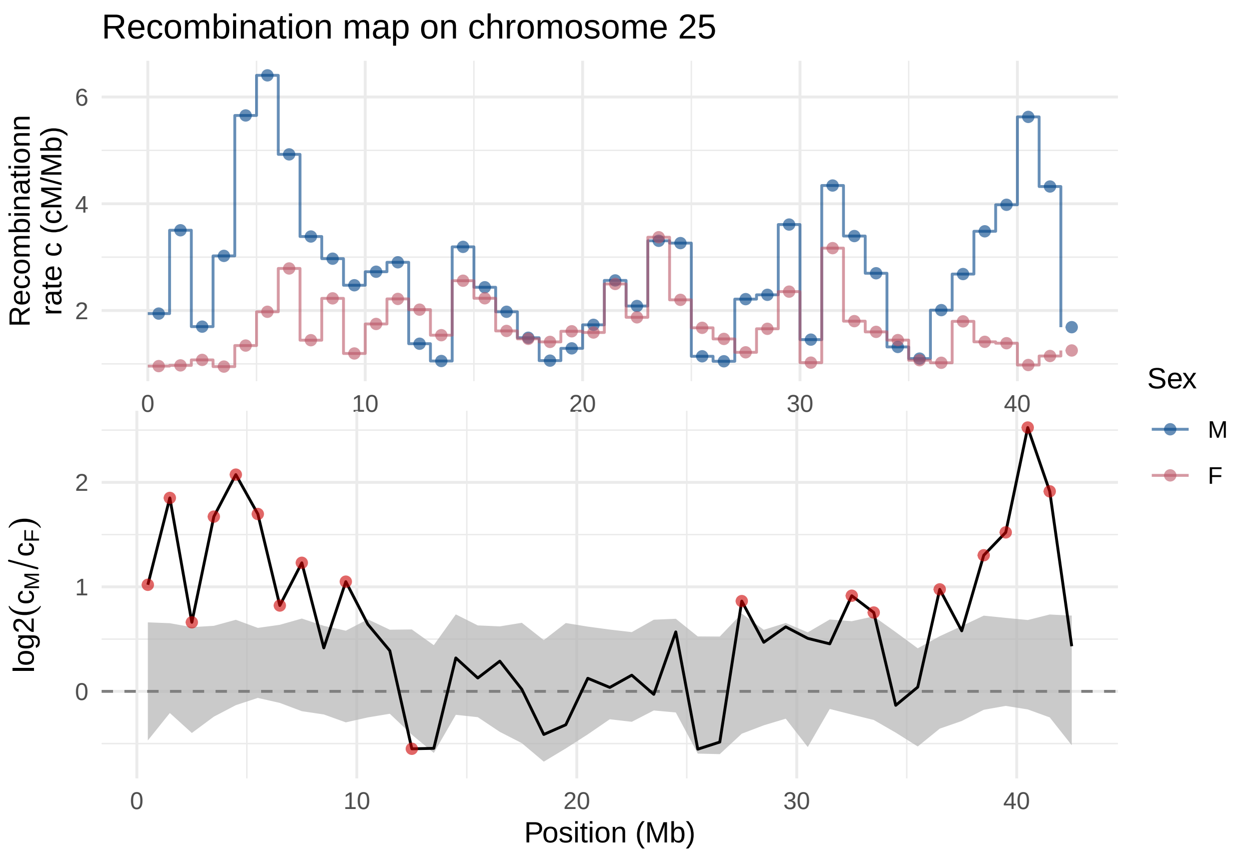
Recombination map on chromosome 25
Recombinationn
rate c (cM/Mb)
6
4
2
0
10
20
30
40
Sex
M
F
log2(c_M/c_F)
2
1
0
Position (Mb)

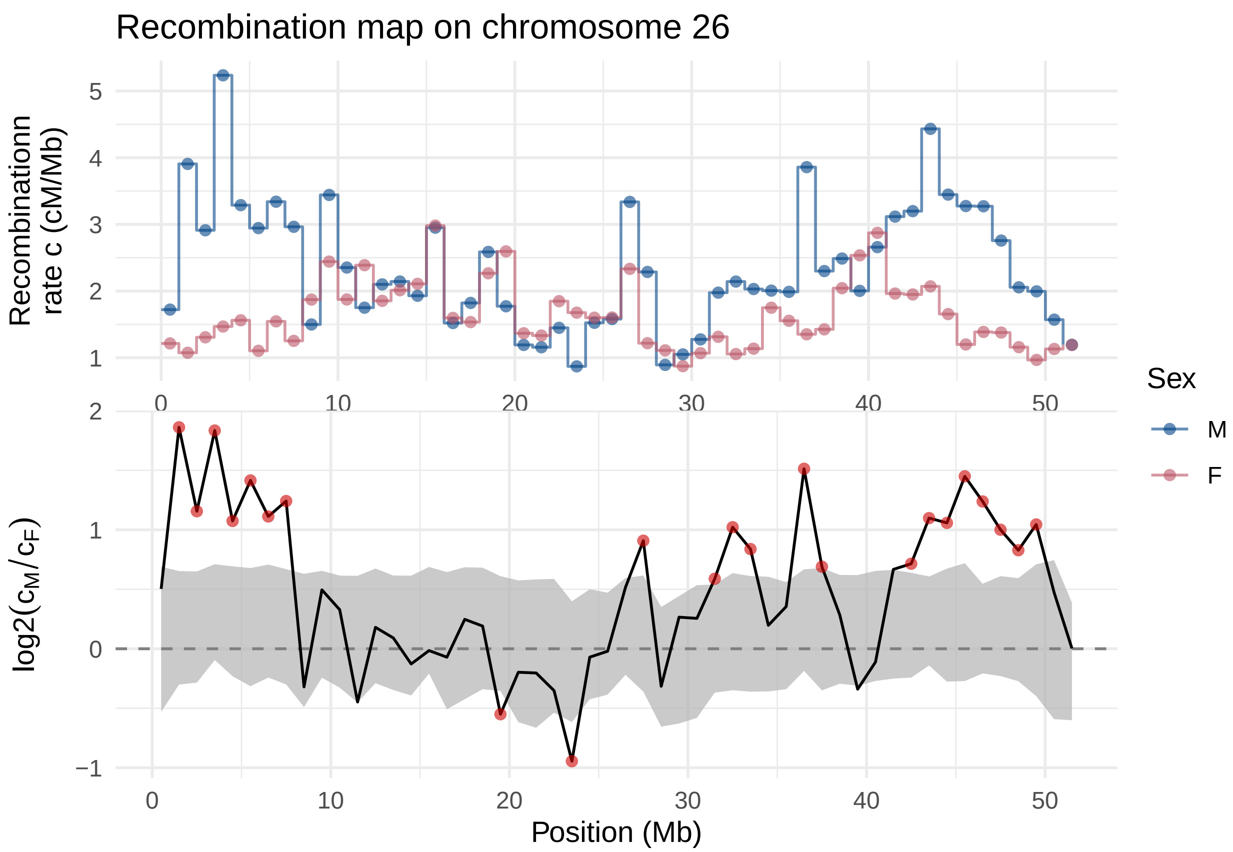
Recombination map on chromosome 26
Recombinationn rate c (cM/Mb)
log2(c_M/c_F)
Position (Mb)
Sex
M
F

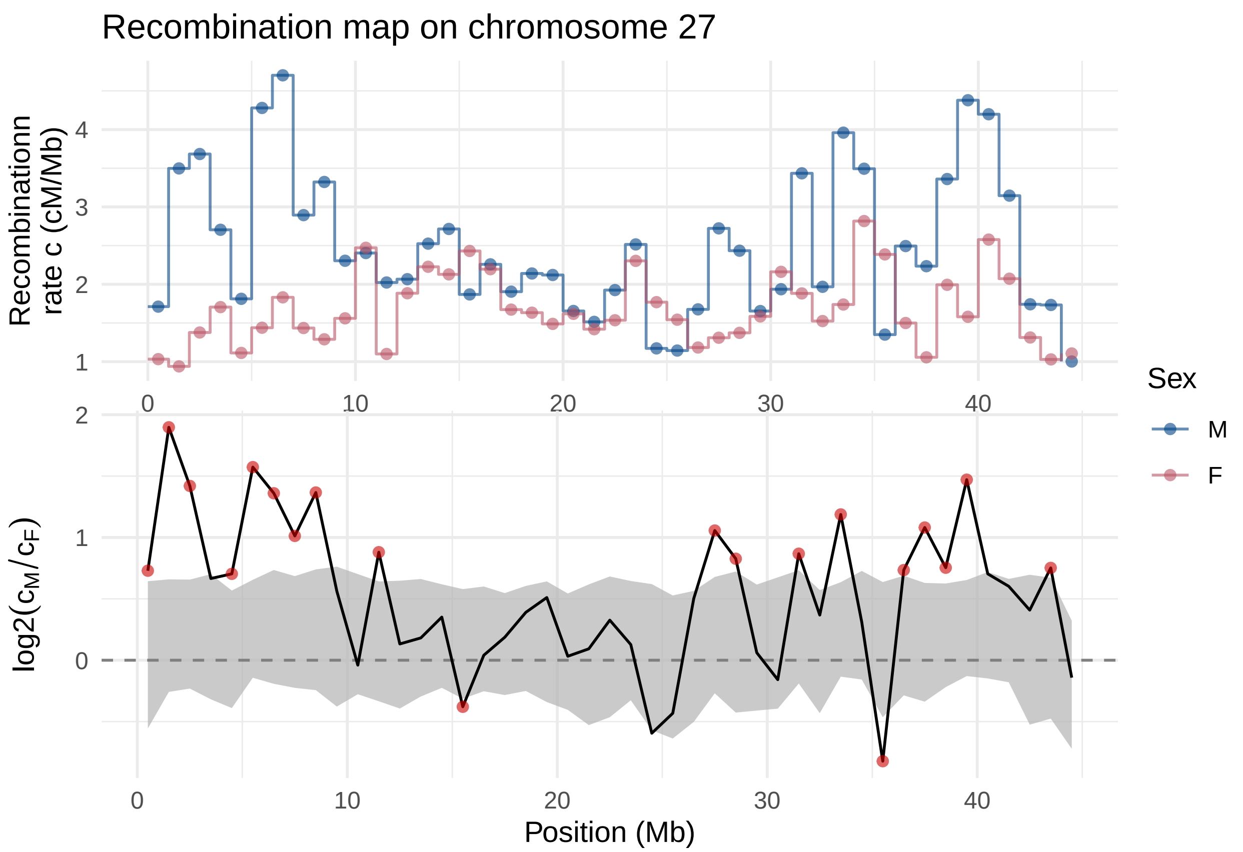

Recombination map on chromosome 27
Recombinationn
rate c (cM/Mb)
log2(c_M/c_F)
Position (Mb)
Sex
M
F

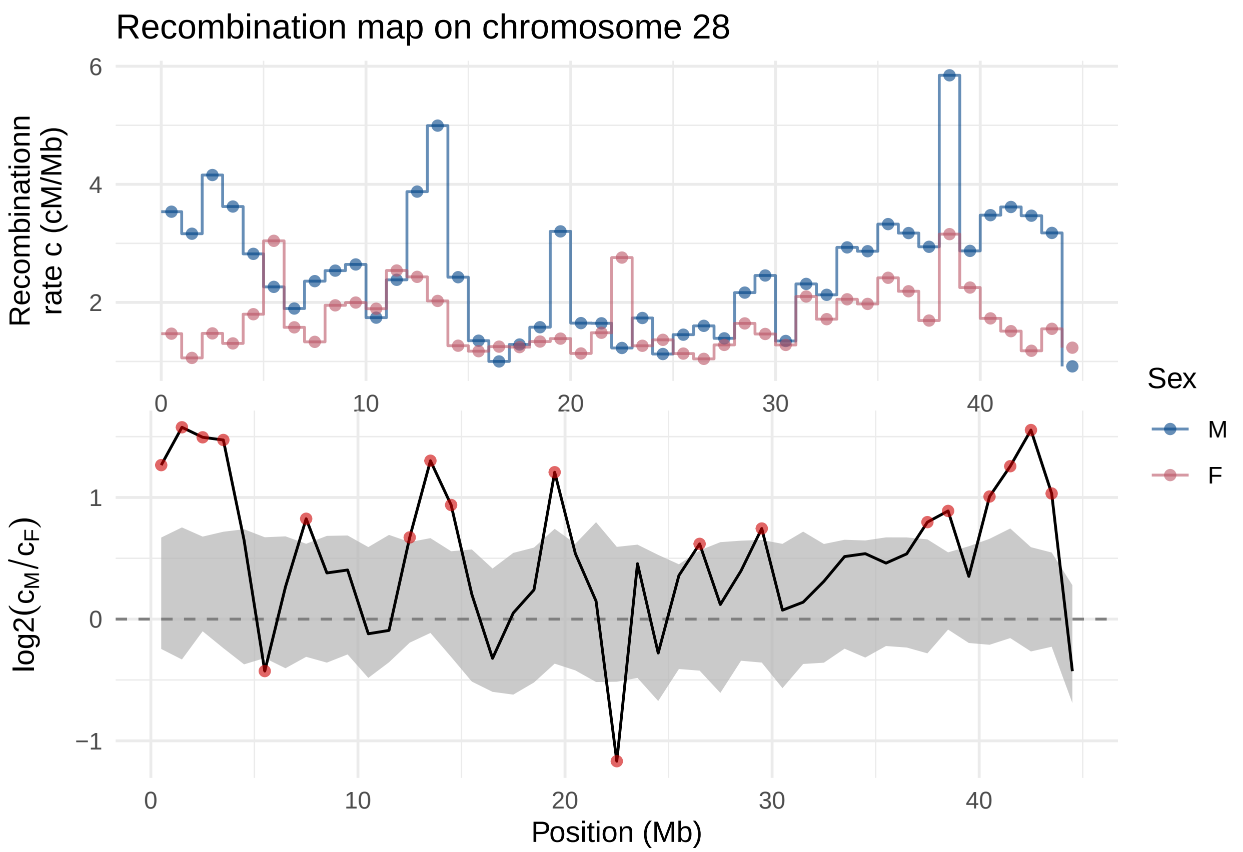

Recombination map on chromosome 28
Recombinationn rate c (cM/Mb)
6
4
2
0
10
20
30
40
Sex
M
F
log2(cM/cF)
1
0
−1
0
10
20
30
40
Position (Mb)

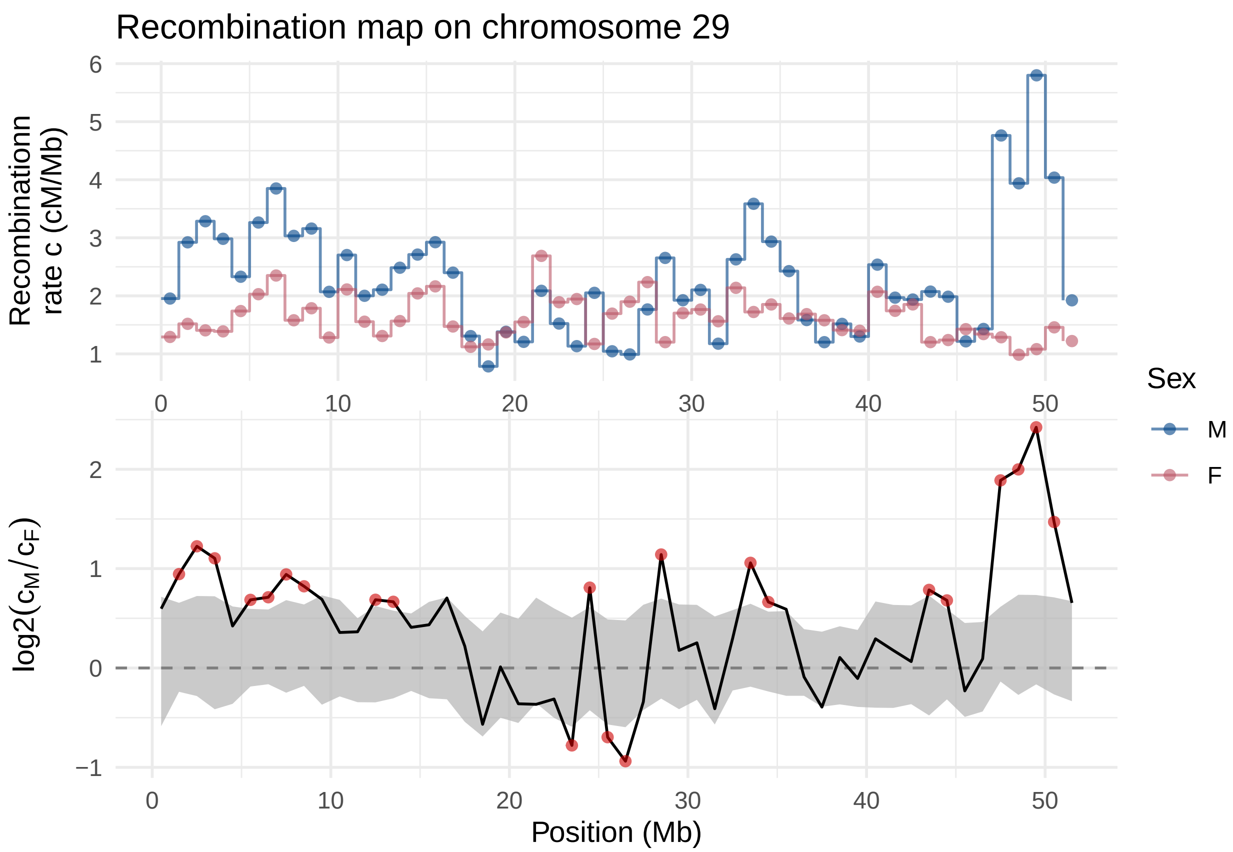

Recombination map on chromosome 29
Recombinationn rate c (cM/Mb)
6
5
4
3
2
1
0
10
20
30
40
50
Sex
M
F
log2(c_M/c_F)
2
1
0
−1
Position (Mb)
